# The novel circSLC6A6/miR-1265/C2CD4A axis promotes colorectal cancer growth by suppressing p53 signaling pathway

**DOI:** 10.1186/s13046-021-02126-y

**Published:** 2021-10-16

**Authors:** Zeyin Rong, Zai Luo, Zhongmao Fu, Pengshan Zhang, Tengfei Li, Jianming Zhang, Zhonglin Zhu, Zhilong Yu, Qi Li, Zhengjun Qiu, Chen Huang

**Affiliations:** 1 Department of General Surgery, Shanghai General Hospital, Shanghai Jiaotong University School of Medicine, 100 Hai Ning Road, Hongkou District, Shanghai, 200080 China; 2 Department of Medical Oncology, Shuguang Hospital, Shanghai University of Traditional Chinese Medicine, Shanghai, China; 3 Academy of Integrative Medicine, Shanghai University of Traditional Chinese Medicine, Shanghai, China

**Keywords:** Colorectal cancer, C2CD4A, p53, circSLC6A6, miR-1265

## Abstract

**Background:**

Colorectal cancer (CRC) is one of the most frequent malignancy and a leading cause of cancer-related deaths. Therefore, further researches are required to identify novel and more effective diagnoses and to identify molecular targets in treatment of CRC.

**Methods:**

C2CD4A expression in CRC tissues and cell lines was detected by qRT-PCR and western blot. The biological functions of C2CD4A were performed both in *vitro* and in *vivo*. Western blot, cDNA array, IP-MS, Co-immunoprecipitation assay, and Ubiquitination assay were used to analyze the interaction between C2CD4A and p53. Bioinformatics analysis, FISH, RNA sequencing, luciferase reporter assay, RNA immunoprecipitation, RNA pull-down and rescue experiments, were deployed to detect upstream regulation mechanism of C2CD4A.

**Results:**

C2CD4A was elevated in CRC tissues compared with adjacent normal colorectal tissues. C2CD4A knockdown significantly promoted cell apoptosis and with inhibited proliferation in *vitro*, and tumorigenicity in *vivo*, whereas C2CD4A overexpression led to opposite effects. Moreover, circSLC6A6 was upregulated and shown to positively regulate C2CD4A expression via sponging miR-1265. Fundamentally, C2CD4A inhibited p53 signaling pathway through interacting with p53 and increasing its ubiquitination and degradation.

**Conclusion:**

Our results identified that circSLC6A6/miR-1265/C2CD4A axis, which was involved in CRC via the p53 signaling pathway, may serve as a therapeutic target for CRC.

**Supplementary Information:**

The online version contains supplementary material available at 10.1186/s13046-021-02126-y.

## Background

Colorectal cancer (CRC), as one of the most common neoplasms, ranks third most frequently diagnosed cancer for males and second for females and is a leading cause of cancer death worldwide [1]. Despite numerous clinical endeavors for early diagnosis and appropriate treatments of CRC in the past few decades, the prognosis of CRC patients was still less desirable, especially in advanced-stage [2]. Hence, further research is urgently required before final goal of working out novel and effective diagnostic methods and identifying precise molecular targets of CRC.

C2CD4A (C2 calcium dependent domain containing 4A) belongs to the C2CD4 family, which included 3 members, namely C2CD4A, C2CD4B and C2CD4C. Due to its high sequence similarity and coding cells nuclear protein, it was also known as the sequence similarity family 148A (FAM148A) or nuclear localization factor 1 (NLF-l) [3, 4]. The C2CD4A gene is located on the human chromosome long arm 15q 22.2, which contains 3941 base pairs, 1 intron and 2 exons. Encoded a protein containing a calcium-dependent lazy C2 domain, C2CD4A gene is a nuclear protein with a relative molecular mass of 39KDa. A slice of change in C2 domain coming along with the variation in C2CD4A gene would affect its encoded protein structure and function. Genome-wide association studies suggested that C2CD4A was highly associated with diabetes and was rarely found in tumor cases [5]. Namely, there were research gap in C2CD4A’s role in regulating the tumorigenesis of CRC and its mechanism in affecting tumor progression.

P53, a major tumor suppressor gene, was essential for genome integrity and stability [6]. As a vital gatekeeper, p53 was mutated in all sorts of cancers for which it would inhibit cell proliferation, induce cell apoptosis, cell senescence, block cell metastasis and regulate energy metabolism by p53’s transcriptional activities [7]. MDM2, a transcriptional target gene of p53, possessed intrinsic E3 ubiquitin ligase activity, was directly bound to p53 and mediated its ubiquitin-dependent proteolysis through its N-terminal, and then formed a negative feedback loop with p53 [8]. Thus, direct or indirect inhibition of the MDM2 activity was crucial for p53 function. Given the critical role of p53 inactivation in the development of CRC, it was significantly important to figure out the molecular mechanism by which p53 was dysregulated in CRC. Our study unveiled that C2CD4A might interact with p53 to increase p53 protein ubiquitin-degradation and thus facilitated the growth of CRC.

Circular RNAs (circRNAs) are defined as an intriguing one in noncoding RNAs (ncRNAs), which are formed by back-splicing or exon skipping and are characterized as covalently closed loop structures with neither 5’ to 3’ polarity nor a polyadenylated tail [9]. CircRNAs are insensitive to multiple exonuclease and have displayed a stable state due to its peculiar structure with the potential to be an ideal biomarker for diagnosis of cancer [10]. And it has a particular cell and tissue type or developmental stage-specific expression pattern in eukaryote, and has been proven to take part in the pathology of different diseases [11]. The specificity of the circRNA structure determines its specific biological functions, such as functions in regulation of gene transcription, and protein translation [12]. Relationships between circRNAs and diseases, especially in cancers, have recently been reported, including ones related to gastric cancer, bladder cancer, liver cancer and CRC [13–16]. In this study, we identified an abnormally upregulated circRNA-circSLC6A6 (hsa_circ_0004705) in CRC cells and tissues, which was significantly associated with CRC patients prognosis. Importantly, circSLC6A6 could bind to miR-1265 and promote the growth of CRC by increasing the expression of C2CD4A protein and facilitating the degradation of p53 protein. Therefore, our research was the first uncovered the novel circSLC6A6/miR-1265/C2CD4A/p53 axis in the CRC growth and its molecular mechanism to provide a promising therapeutic targets of CRC.

## Materials and methods

### Patients and tissue samples

108 paired CRC and adjacent normal tissues were collected from Shanghai General Hospital between 2013 and 2014, and were paraffin embedded for the tissue microarray (TMA) construction (the final TMA contained 106 CRC tissues and 106 adjacent normal tissues). Meanwhile, sixty-three pairs of human CRC fresh tissues and adjacent normal tissues were collected from Shanghai General Hospital after radical surgical resection between 2015 and 2017. After resection, the tissues were transported in liquid nitrogen and stored at -80°C refrigerator for RNA and protein extraction. No patients had received chemotherapy and radiotherapy before surgery. The detailed clinicopathological feature was confirmed by at least two pathologists according to the American Joint Committee on Cance (AJCC). Our research was approved by the Ethics Committee for Clinical Research of Shanghai General Hospital.

### Animal experiments

Male BALB/c athymic nude mice ( 4 weeks old) were randomly divided into 3 groups (five mice per group). Stable transfected RKO cells (5×10^6^) were subcutaneously injected into subscapular in each group to establish the CRC xenograft model. Tumor size was measured every week to monitor tumor growth. The tumor volumes were calculated using the formula: 0.5×length×width^2^. All mice were sacrificed at 38 days; all tumors were surgically removed and weighed. After fixed in 4% paraformaldehyde, all tumors were embedded and sliced for IHC staining. All animal experiments were approved by the Institutional Animal Care of Shanghai General Hospital.

### Luciferase reporter assay

Luciferase plasmids (pGL3-Firefly-Renilla containing circSLC6A6 sequence or Mutant sequence, pGL3-Firefly-Renilla containing C2CD4A 3’-UTR sequence or Mutant sequence) were purchased from GenePharma (Shanghai, China) and were co-transfected with miR-1265 mimics or inhibitor to indicated cells using Lipofectamine^TM^ 2000. In short, the transfected cells were lysed with 100μl of passive lysis buffer and the supernatant was collected after centrifuge. Cell samples (20μl) and 100μl of LARII were added into 96-well luminometer plates, followed by firefly luciferase activity detection. Then, 100μl of Stop & GloR reagent was added into 96-well luminometer plates to detect Renilla luciferase activity. The ratio of firefly luciferase/Renilla luciferase activity was used to detect the effects of miR-1265 on luciferase reporter plasmids. All experiments were independently performed in triplicate.

### Fluorescence in situ hybridization (FISH)

FISH assay was performed to locate circSLC6A6 using a Cy3-labeled probe and to locate miR-1265 using a FAM-labeled probe in HCT8 and RKO cells, respectively. The Cy3-labeled circSLC6A6 probe and FAM-labeled miR-1265 probe were designed and synthesized by GenePharma (Shanghai, China). Cells were seeded in 35-mm glass bottom dishes with 10-mm microwells. After washing with PBS and being fixed with anhydrous ethanol, the cells were treated with 100μl of 0.1% Triton-100 at room temperature for 15 min. The cells were hybridized with 5μl of probe in hybridization buffer (10% dextran sulfate, 40% formamide, 4×saline-sodium citrate (SSC), 1×Denhardt's solution, 1000 mg/ml sheared salmon sperm DNA, 10 mM DDT, 1000 mg/ml yeast transfer RNA) at 37°C overnight. Then, cells were continuously washed and dyed with 100μl of DAPI for 20 min. Then, confocal laser scanning microscopy was used to observe the staining. Simultaneously, FISH assay was also performed in TMA, which contained 106 paired CRC samples by Cy3-labeled miR-1265 (Boster, Shanghai). The staining scores were evaluated by two independent pathologists to avoid bias as previously described in our published study [17]. Final, the samples scores were divided into two groups: the high expression group (4-8) and the low expression group (0-3). The sequences of circSLC6A6 and miR-1265 probe for FISH and TMA were listed in Additional file 2: TableS3.

### Immunoprecipitation and Mass Spectrometry (IP/MS)

293 T cells transfected with Flag-C2CD4A were lysed in RIPA buffer (20 mM Tris-HCl (pH = 8), 137 mM NaCl, 0.5% Triton X-100, 2 mM EDTA) and protease inhibitor cocktail (Sangon Biotech, Shanghai, China). Cell lysates were incubated with Flag-M2 agarose beads (Sigma) overnight and eluted by Flag peptides (Sigma). The Flag peptide elution was resolved on SDS-PAGE and Coomassie blue stained. Lysates from 293 T cells transfected with control Flag-vector were served as control. Protein bands specific to the Flag-C2CD4A transfection were digested with trypsin and analyzed by mass spectrometry for protein identification.

### Co-immunoprecipitation assay (Co-IP) and ubiquitination assay

Protein extracts were immunoprecipitated with Protein A+G Agarose beads (Beyotime Biotechnology, Shanghai, China) using appropriate antibodies or control IgG (Sigma, USA). Then, the protein of immunocomplexes were subject to immunoblotting analysis with indicated antibodies. For ubiquitination assay, 293 T cells were co-transfected with Flag-C2CD4A, HA-P53 and His-ubiquitin. Alternatively, 293 T cells were co-transfected with sh-C2CD4A, HA-P53 and His-ubiquitin. 24 hour later, 293 T cells were treated with MG132 (10μM) for 8 hours to inhibit proteasomal degradation. The transfected cells were lysed and centrifuged. Then the supernatants were pulled-down by incubation with Ni-NTA beads, and the protein of immunocomplexes was immunoblotted using anti-HA antibody.

### RNA immunoprecipitation (RIP)

RIP was performed by using Magna RIP™ RNA-binding protein immunoprecipitation kit (Millipore, Billerica, MA) according to manufacturer’s protocol. The Ago2-RIP experiments were conducted in RKO cells transiently overexpressing miR-1265 mimics or miR-NC. 48 h later, approximately 1×10^7^ RKO cells were lysed in complete RNA lysis buffer. Then, RKO cell lysates were incubated with RIP immunoprecipitation buffer containing magnetic beads conjugated with human anti- Ago2 or negative control IgG (Millipore, Billerica, MA, USA) at 4°C overnight. Each sample was digested with Proteinase K and then immunoprecipitated RNA were analyzed by qRT-PCR to test the concentration of circSLC6A6. RIP assay was repeated in triplicate.

### RNA Pull-down assay

A pull-down assay was performed as reported [17]. The biotinylated circSLC6A6 probe and miR-1265 probe were designed and synthesized by GenePharma (Shanghai, China) and the sequences were listed in Additional file 2: TableS4. Briefly, cells were collected, lysed, and sonicated. Probe-coated beads were generated by coincubating the circSLC6A6 and miR-1265 probes with probes-M280 streptavidin dynabeads (Invitrogen, USA) at 25°C for 2h. The cell lysates were incubated with circSLC6A6 and miR-1265 probes or oligo probe at 4°C overnight. After extensively washed, the RNA complexes bound to the beads were eluted and extracted with the RNAisoPlus (TaKaRa, Japan) and measured by qRT-PCR.

### Statistical analysis

All quantitative data were enumerated by a χ2 or Fisher’s exact test. The associations were analyzed by Pearson’s test. Comparisons between different groups were analyzed using a paired or unpaired t-test. Survival curves were drawn by Kaplan-Meier method. The SPSS 23.0 software was deployed for statistical analysis. P < 0.05 was statistically significant in all tests.

A complete description of the methods, including cell culture and culture conditions, RNA extraction, gDNA extraction, and quantitative real-time polymerase chain reaction (qRT-PCR), uucleic acid electrophoresis and RNase R treatment, Immunohistochemistry (IHC), cell proliferation assay, apoptosis analysis, western blot analysis, the cancer genome atlas (TCGA) database, GEPIAdataset, Oncomine database, cDNA array analysis, transfection, oligonucleotides and plasmids are available in Additional file 1: Supplementary materials and methods.

## Results

### C2CD4A is frequently upregulated in CRC

**Fig. 1:**
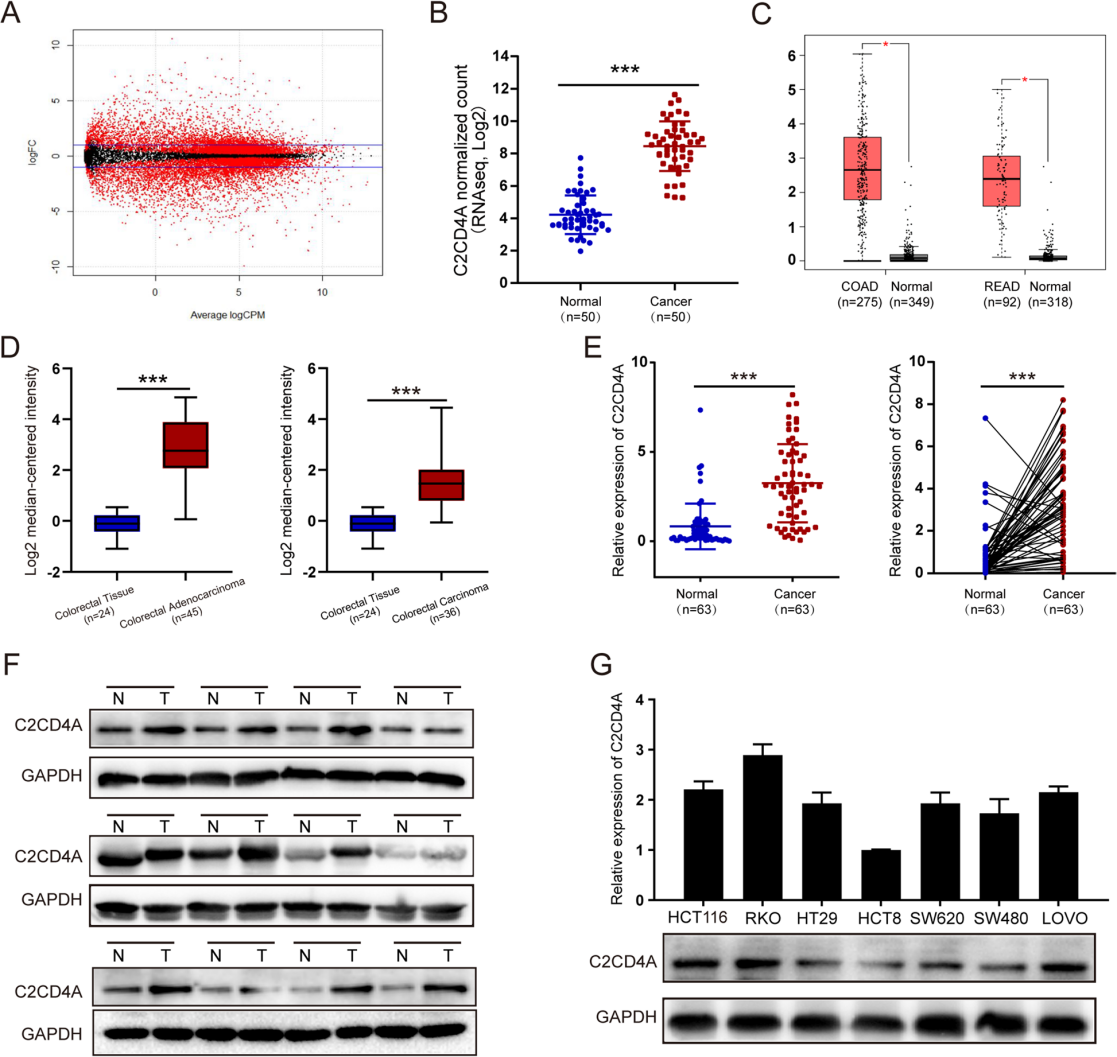
C2CD4A expressions in CRC tissues and cell lines. **a** The scatter diagram showed the differential mRNA expression among 50 CRC cancer samples and paired adjacent normal colorectal mucosae samples from TCGA database. **b** Relative C2CD4A mRNA expression in 50 paired CRC tissues and paired adjacent normal colorectal mucosae from TCGA database. **c** The expression of C2CD4A was significantly higher in CRC tissues than in normal tissues in the GEPIA dataset. (*P* < 0.05 as calculated by t-test). **d** C2CD4A expression in CRC specimens from the Oncomine datasets (Skrzypczak Colorectal Statistics, 2010). **e** C2CD4A expression was elevated in 63 fresh frozen CRC tissues samples compared with paired adjacent normal mucosae by using qRT-PCR. **f** C2CD4A protein expressions in representative paired CRC tissue samples were detected by western blot. **g** qRT-PCR and western blot helped to detect the expression of C2CD4A in CRC cell lines. (**P < 0.01, ***P < 0.001)

Through analysis of the CRC clinical samples from the Cancer Genome Atlas (TCGA) database, there were 461 CRC samples with RNAseq and RNAseqV2 data, in which 50 paired samples were with pathological data and RNAseq data (41 paired samples colon adenocarcinoma samples and 9 paired rectum adenocarcinoma samples). Based on the above 50 paired CRC samples, the volcano plots showed CRC related differentially expressed genes were screened (Fig. 1A; Additional file 3: TableS6). Then, we found that C2CD4A (C2 calcium dependent domain containing 4A) expression was significantly higher in CRC tissues than in the paired normal colorectal tissues (19.869 fold change, *P* < 0.001) (Fig. 1B), and has never been investigated in tumors. GEPIA dataset also comfirmed that C2CD4A was higher in CRC tissues than in normal tissues (Fig. 1C). On the basis of Oncomine database (www.oncomine.org), C2CD4A was also significantly higher in 45 colorectal adenocarcinoma and 36 colorectal carcinoma compared with normal tissues (Skrzypczak Colorectal Statistics, 2010), respectively (Fig. 1D). To further confirm the variation of the expression of C2CD4A in CRC tissues, qRT-PCR analysis showed the upregulation of C2CD4A in 63 pairs of fresh frozen CRC tissues compared to adjacent noncancer tissues (Fig. 1E). Western blot results further confirmed that C2CD4A was upregulated in randomly selected 12 pairs of fresh frozen CRC tissues (Fig. 1F). Meanwhile, C2CD4A mRNA and protein expression were also detected in seven CRC cell lines. C2CD4A was presented with the highest expression in RKO cell and with the lowest expression in HCT8 cell, respectively (Fig. 1G). Hence , we chosen RKO and HCT8 cells for subsequent functional assays.

### C2CD4A promoted CRC cell growth in *vitro* and in *vivo*

**Fig. 2:**
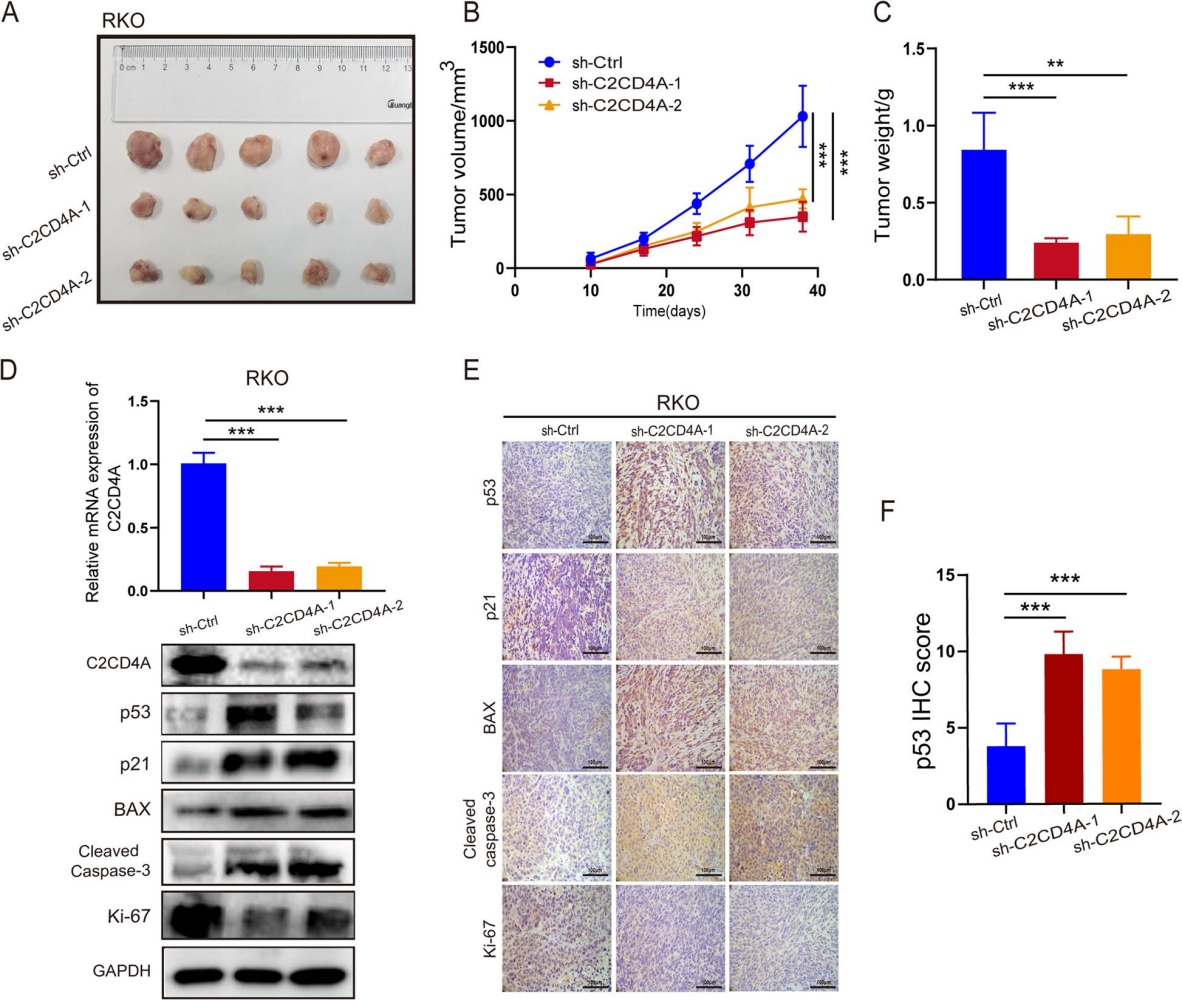
C2CD4A promoted CRC cell growth in *vivo*. **a** Image of different groups subcutaneous xenograft tumors of RKO cells was shown (five mice per group). **b** The volume of subcutaneous xenograft tumors were measured every week for five weeks. **c** The average final tumors weight was shown. **d** qRT-PCR and western blot were performed to detect mRNA and protein level of C2CD4A in tumors. And the protein expression levels of p53, p21, BAX, Cleaved-caspase3 and Ki-67 were shown using western blot. **e** IHC staining of p53, p21, BAX, Cleaved-caspase3 and Ki-67 from the indicated tumors. **f** Quantification of p53 expression was determined by IHC scores. Date was shown as mean ± SD with three experiments. (*P < 0.05, **P < 0.01, ***P < 0.001)

To explore the biological functions of C2CD4A in CRC, knockdown and overexpression vectors (sh-Ctrl, sh1, sh2 and pLVX-Ctrl, pLVX-C2CD4A) were respectively transfected with lentiviral vectors to generate stable knockdown or overexpression cell lines. The effects of knockdown and overexpression on C2CD4A were confirmed by qRT-PCR and western blot analysis (Additional file 4: Fig. S1A). Subsequently, CCK-8 assay, EdU assay and clone assay results revealed that the growth rate of RKO cell was noticeably attenuated in the downregulated C2CD4A expression (Additional file 4: Fig. S1B, C and D). Overexpression of C2CD4A led to opposite effects in HCT8 cells (Additional file 4: Fig. S1B, C and D). Next, flow cytometry indicated that downregulated expression of C2CD4A increased the percentage of apoptotic RKO cells, whereas upregulated expression of C2CD4A decreased the percentage of apoptotic HCT8 cells (Additional file 4: Fig. S1E). Based on in *vitr*o findings, we next tested the role of C2CD4A in *vivo*. A xenograft model was constructed by subcutaneous injection of tumor cells in nude mice. The results showed that the tumor volumes were significantly diminished in knockdown groups (Fig. 2A and B). Furthermore, the mean tumor weights of the knockdown groups were significantly lower than those of the control groups (Fig. 2C). We then extracted the total RNA and protein from the tumors in different groups. qRT-PCR and western blot confirmed that the expression of C2CD4A were significantly decreased in knockdown groups (Fig. 2D). Likewise, western blot and IHC staining showed that the levels of Ki-67 were obviously decreased by C2CD4A knockdown, while the levels of Cleaved-caspase3 were markedly elevated (Fig. 2D and E). Taken together, the results above suggested that C2CD4A promoted growth and inbibited apoptosis both in *vitro* and in *vivo* in CRC cells.

### C2CD4A promotes CRC cells growth via p53 signaling pathway and interacts with p53

**Fig. 3:**
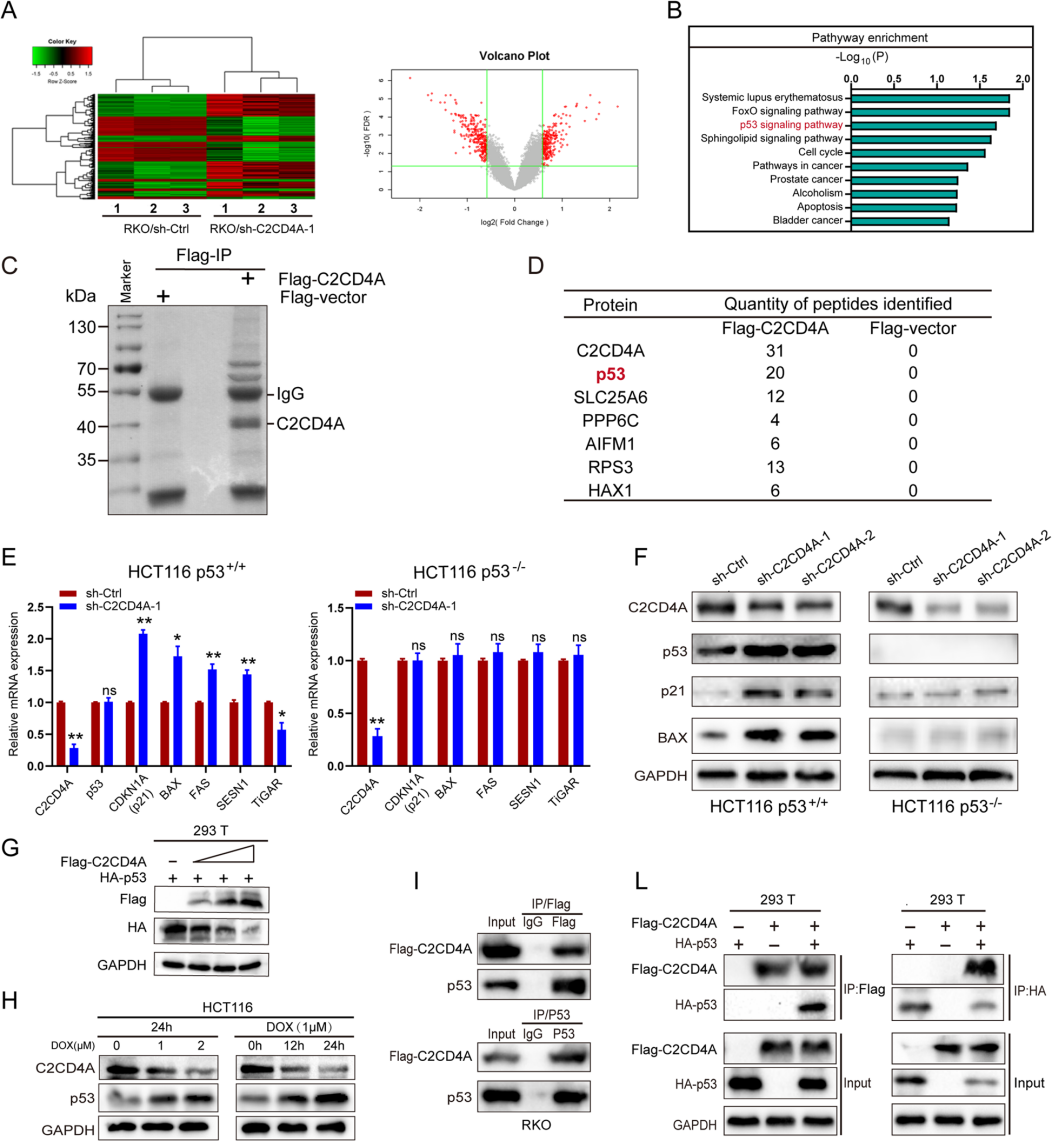
C2CD4A promoted CRC cells growth via p53 signaling pathway and interacted with p53. **a** The heatmap and scatter diagram showed the differential expressed mRNA between RKO/sh-C2CD4A (KD-1, 2, 3) and RKO/sh-Ctrl cells (NC-1, 2, 3) screened by human cDNA microarray. **b** Top 10 pathways of KEGG analysis based on DEGs. **c** IP/MS analysis of Flag-C2CD4A-associated proteins. The Flag peptide elution was resolved on SDS-PAGE and Coomassie blue staining. **d** Several proteins related to cell apoptosis identified from Flag-C2CD4A IP/MS analysis. **e** The relative mRNA expressions of C2CD4A, p53, CDKNIA(p21), BAX, FAS, SESN1 and TiGAR were detected using qRT-PCR. **f** The levels of C2CD4A, p53, CDKNIA(p21) and BAX protein expression were detected using western blot. **g** 293 T cells were co-transfected with Flag-C2CD4A and HA-P53 at various concentrations, and then Flag and HA expression were detected using western blot. **h** HCT116 cells were treated with Dox at 1, 2 μM for 24 h, and treated at 1 μM for 12, 24 h. C2CD4A and p53 expressions were detected using western blot. **i** The lysates of RKO cells were transfected with Flag-C2CD4A followed by immunoprecipitated using anti-IgG, anti-Flag or anti-p53 antibodies, and immunoprecipitation products were analyzed by western blot with indicated antibodies. **l** 293 T cells were co-transfected with Flag-C2CD4A and HA-p53. Immunoprecipitation was carried out using anti-Flag or anti-HA antibody, and immunocomplexes were detected using indicated antibodies. (ns showed no significance. *P < 0.05, **P < 0.01)

Considering our finding that C2CD4A was related to growth, and apoptosis of CRC cells, we aimed to investigate C2CD4A-relevant molecular mechanisms in the process of CRC. We analyzed the correlation between C2CD4A and other genes in CRC using a cDNA array to identify differentially expressed genes (DEGs) between three pairs knockdown and control cells (RKO/sh-C2CD4A-1 and RKO/sh-Ctrl). According to the results of analysis of DEGs regulated by C2CD4A, 232 and 185 genes were upregulated and downregulated (|fold changes| > 1.5, *P* < 0.05), respectively (Fig. 3A), with CDKN1A (p21), BAX, FAS and SESN1 exhibiting the high expression levels and BCL2, Ki67 and TiGAR exhibiting the low expression levels (Additional file 2: TableS5). P21, BAX, FAS, SESN1, and TiGAR are all the down-stream genes of p53 transcriptional targets [18, 19]. KEGG pathway analysis of these DEGs revealed that p53 signaling pathway was significantly enriched among the top 10 enrichment-regulated pathways (Fig. 3B). Moreover, we performed the IP/MS to identify key factors associated with C2CD4A. 293 T cells transfected with Flag-C2CD4A was immunoprecipitated by Flag-M2 agarose beads and the interacting proteins were visualized by Coomassie blue staining after electrophoresis and identified by mass spectrometry (Fig. 3C). We found that one interacting protein turned out to be p53 among the Flag-C2CD4A-interacted proteins associated with cell apoptotic (Fig. 3D). As the p53 signaling pathway was of significant influence in the regulation of tumor progression [20], it was inferred that C2CD4A might promote the CRC growth by repressing the p53 signaling pathway based on our preliminary results. Thus, we further analyzed the correlation between the expression of C2CD4A and p53 down-stream genes. To verify this hypothesis, we measured the expression variation of p53 down-stream genes in RKO, HCT116 p53^**+/+**^ and HCT116 p53^**−/−**^ cells with stable C2CD4A knockdown. The qRT-PCR results showed that C2CD4A knockdown increased p21, BAX, FAS and SESN1 expressions, and decreased TiGAR expression, while the level of p53 mRNA expression remained unchanged in RKO and HCT116 p53^**+/+**^ cells (Fig. 3E, Additional file 5: Fig. S2A). Likewise, western blot results showed that C2CD4A knockdown had increased the expressions of p53, p21, and BAX level in HCT116 p53^**+/+**^ cells (Fig. 3F). But there is no change in HCT116 p53^**−/−**^ cells (Fig. 3F). In addition to that, the expression of p53, p21 and Bax were significantly elevated in tumors with C2CD4A knockdown in the CRC xenograft mice model using western blot and IHC (Fig. 3D, E). More impotantly, the knockdown of C2CD4A obviously increased the expression of p53 protein (Fig. 3E, F). Collectively, these data suggested C2CD4A might promote the CRC growth and proliferation by repressing the p53 signaling pathway.

We further focused on C2CD4A regulating p53 and its molecular mechanism according to the results of cDNA array and IP/MS. To address the relationship between C2CD4A and p53, 293 T cells were co-transfected with Flag-C2CD4A and HA-p53 at various concentrations, and then the level of p53 expression was detected. P53 expression decreased with incremental dose of C2CD4A, showing a dose-dependent manner between C2CD4A and p53 (Fig. 3G). Doxorubicin (DOX) is a DNA damaging agent that has been conformed to increase p53 expression [21]. We used different concentrations of DOX to induce p53 expression, and then observed variations of C2CD4A expression in HCT116 cells. The level of p53 expression gradually elevated with the increasing concentration of DOX, whereas the level of C2CD4A expression gradually decreased (Fig. 3H). Therefore, it manifested a significantly concentration-dependent manner between C2CD4A and p53 expression. Above results indicated that C2CD4A and p53 expression were negatively correlated in a dose-dependent manner. Furthermore, we carried out endogenous immunoprecipitation assay to confirm that Flag-C2CD4A interacted with p53 in RKO cells (Fig. 3I). Next, exogenous Flag-C2CD4A and HA-p53 were co-transfected into 293 T cells, and then Flag-C2CD4A or HA-p53 were respectively immunoprecipitated (Fig. 3L). Taken together, these data demonstrated that interacted with p53.

### C2CD4A decreased half-life of p53 and promoted ubiquitin degradation of p53 by enhancing the MDM2-p53 interaction

**Fig. 4:**
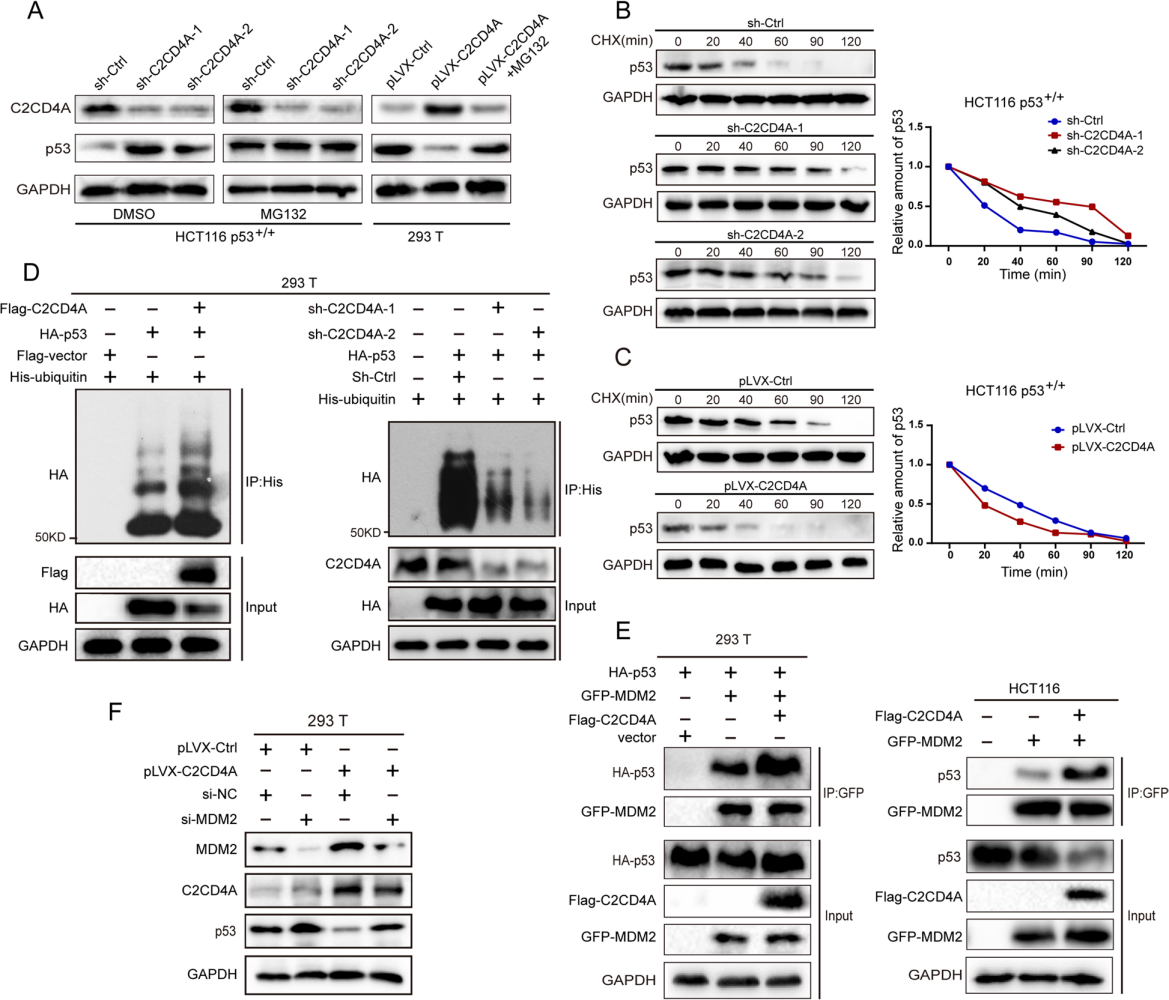
C2CD4A decreased half-life of p53 and promoted ubiquitin degradation of p53 by enhancing the MDM2-p53 interaction **a** C2CD4A-knochdown HCT116 p53+/+ cells were treated with DMSO and MG132. The treatment of DMSO served as a control. 293 T cells, with stable overexpressed C2CD4A, were treated with MG132. The level of C2CD4A and p53 expression were detected using western blot. **b**, **c** C2CD4A-knochdown HCT116 p53^+/+^ cells (**b**), C2CD4A-overexpression (**c**) and control HCT116 p53^+/+^ cells were transfected with CHX and harvested at indicated points after CHX treatment, respectively. The level of C2CD4A and p53 expression were detected using western blot. **d** 293 T cells were co-transfected with Flag-C2CD4A, HA-p53, His-ubiquitin or vector. His was immunoprecipitated using anti-His antibody, and p53 ubiquitination was detected using anti-HA antibody (left). 293 T cells were co-transfected with His-ubiquitin, sh-C2CD4A-1, sh-C2CD4A-2, HA-p53 or vector. His was immunoprecipitated using anti-His antibody, and P53 ubiquitination was detected using anti-HA antibody (right). co-immunoprecipitation revealed the polyubiquitination of HA-p53. Co-IP assays revealed the polyubiquitination of HA-p53. **e** 293 T and HCT116 cells were co-transfected with the indicated plasmids, and Co-IP assays were performed using anti-GFP antibody to detect the interaction between p53 and MDM2. **f** The p53, MDM2, C2CD4A expressions were detected in the tranfected 293 T cells using western blot

Former results demonstrated that C2CD4A could bind with p53 and decrease p53 protein expression. It was speculated that C2CD4A had affected the proteolysis of p53. HCT116 p53^+/+^ cells with stable C2CD4A knockdown were treated with MG132, a specific proteasome inhibitor. Then, the results form western blot analysis indicated that the increased p53 protein expression caused by C2CD4A knockdown were mainly reversed by MG132 (Fig. 4A). Additionally, 293 T cells, with stable overexpressed C2CD4A, were also treated with MG132. The results from western blot analysis showed that the decreased expression of p53 caused by C2CD4A overexpression were partially reversed by MG132 (Fig. 4A). Above results indicated that C2CD4A medicated the downregulation of p53 by proteasomal degradation. Then, HCT116 p53^+/+^ cells with stable C2CD4A knockdown were treated with CHX. The results from time-dependent protein degradation rate assays showed that knockdown of C2CD4A in HCT116 p53^**+/+**^ cells significantly decreased the p53 degradation rate, and extended the half-life of endogenous p53 protein (Fig. 4B). Meanwhile, we also successfully overexpressed C2CD4A in HCT116 p53^+/+^ cells (Additional file 5: Fig. S2B), and C2CD4A overexpression shortened the half-life of endogenous p53 protein (Fig. 4C). The results indicated that C2CD4A was relevant to stability of p53 protein. Ubiquitin-proteasome was a highly effective protein degradation pathway in eukaryotic cells [22]. It was widely acknowledged that ubiquitin-mediated degradation through the proteasomal pathway was canonical mechanism for regulating p53 protein homeostasis [23]. Further, it was discussed that whether C2CD4A was of influence on p53 ubiquitination. Accordingly, p53 poly-ubiquitination was elevated in 293 T cells when transfected with Flag-C2CD4A (Fig. 4D). Conversely, downregulated C2CD4A expression significantly inhibited p53 poly-ubiquitination (Fig. 4D). E3 ubiquitin ligase murine double minute (MDM2) was not only a transcriptional target of p53 but also the most important negative mediator for p53 protein stability by binding and ubiquitinating p53 via proteasomal degradation pathway [24, 25]. To confirm MDM2’s role in stimulating C2CD4A’s down-regulating p53, p53 protein expression level was monitored after that 293 T cells were transfected with HA-p53, GFP-MDM2 and Flag-C2CD4A, as determined by immunoprecipitation. Intriguingly, GFP-MDM2 binding to HA-p53 was remarkably elevated in the 293 T cells with Flag-C2CD4A transfection (Fig. 4D). Further, Flag-C2CD4A coimmunoprecipitated with MDM2 in RKO cells (Additional file 5: Fig. S2C). Concomitantly, Co-IP assay was performed to figure out the interaction among endogenous p53 and GFP-MDM2 in Flag-C2CD4A-overexpressing HCT116 cells. Much more GFP-MDM2 was bound to endogenous p53 when HCT116 cells were transfected with Flag-C2CD4A (Fig. 4E). To further confirm whether C2CD4A mediates p53 reduction through MDM2, 293 T cells, with stable overexpressed C2CD4A, were transfected with siRNAs targeting MDM2 to detect the expression of p53. The results of western blot showed that C2CD4A decreased the expression of p53, but the expression of p53 was not remarkably decreased when the silencing of MDM2 (Fig. 4F). Collectively, this data suggested that C2CD4A increased p53 poly-ubiquitination and thereby promoted its proteasomal degradation by enhancing the MDM2-p53 interaction

### MiR-1265 was downregulated in CRC and suppressed C2CD4A expression by directly binding to the 3’UTR of C2CD4A mRNA

**Fig. 5:**
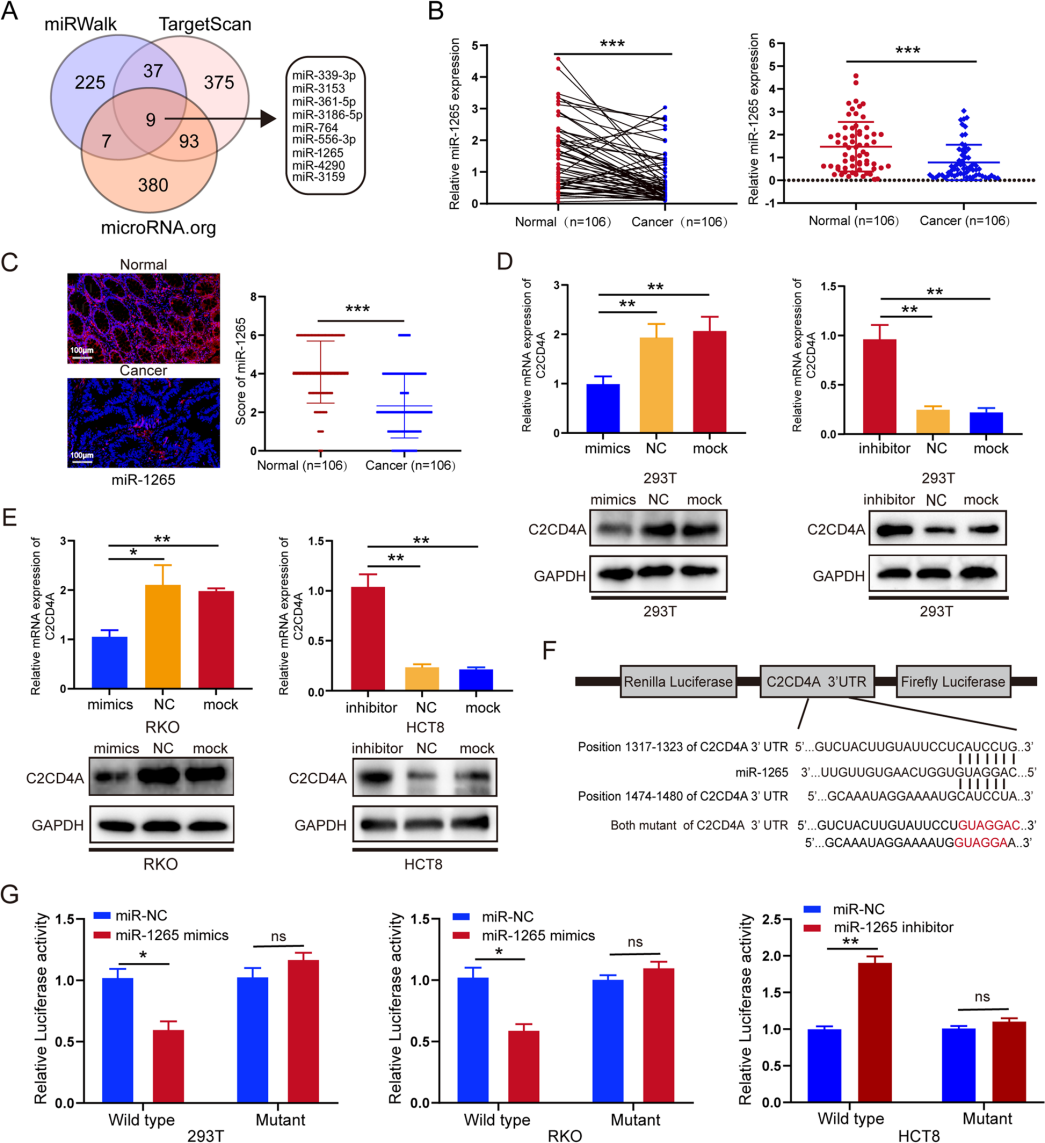
MiR-1265 was downregulated in CRC and suppressed C2CD4A expression by directly binding to the 3’UTR of C2CD4A mRNA. **a** Venn diagram illustrated the overlap of miRNAs detected in the miRWalk, TargetScan and microRNA.org. **b** The relative expression levels of miR-1265 were independent experiments were performed for each by qRT-PCR in 63 pairs of fresh frozen CRC tissues and adjacent noncancer tissues. **c** The FISH results showed that the level of miR-1265 was lower in CRC tissues than that in adjacent normal mucosae tissues from the TMAs (n = 106). **d**, **e** 293 T, RKO, and HCT8 cells were transfected with mock, miRNA control and miR-1265 mimics or miR-1265 inhibitor; and the expression of C2CD4A were detected by qRT-PCR and western blot. **f** Schematic of C2CD4A 3’UTR wild-type (WT) and mutant (Mut) luciferase reporter vector are shown. **g** Wild type (WT) or mutant C2CD4A 3’-UTR were transfected into 293 T, RKO, HCT8 with miR-1265/NC or miR-1265 mimics or miR-1265/inhibitor. Luciferase reporter assay were performed to detect different groups luciferase activity, respectively. Mean ± SEM was shown for these data. Three independent experiments were performed for each group. (ns showed no significance. *P < 0.05, **P < 0.01, ***P < 0.001)

Accumulating evidences indicated that altered expressions of miRNAs were involved in tumor cell proliferation, apoptosis, metabolism, and metastasis [26]. Based on our previous studies on miRNAs in gastrointestinal tumors [13, 17], it was aimed to further figure out whether the overexpression of C2CD4A in CRC was caused by a specific miRNAs dysregulation. Using bioinformatics prediction tools, such as Targetscan, miRWalk and Microrna.org, among those miRNAs which could bind with 3’UTR of C2CD4A, we preliminarily screened out hsa-miR-339-3p, hsa-miR-3153, hsa-miR-361-5p, hsa-miR-3186-5p, hsa-miR-764, hsa-miR-556-3p, hsa-miR-1265, hsa-miR-4290 and hsa-miR-3159 as potential regulators of C2CD4A (Fig. 5A). For above potential regulators, it was found that hsa-miR-339-3p, hsa-miR-361-5p and miR-1265 had been reported in different tumors [27–30], in which hsa-miR-339-3p and hsa-miR-361-5p were lowly expressed in CRC and had assumed the role of tumor suppressing [31, 32]. Next, we used qRT-PCR to detect the expression of miR-339-3p, hsa-miR-361-5p and miR-1265 in 63 pairs fresh frozen CRC tissues, and found that miR-1265 was significant lower in 63 pairs fresh frozen CRC tissues (48/63, 76.2%) compared with those adjacent noncancer tissues (Fig. 5B, Additional file 6: Fig. S3A). Next, qRT-PCR results indicated that miR-1265 expression was negatively correlated with C2CD4A mRNA expression in the above CRC samples (Additional file 6: Fig. S3B). However, C2CD4A expression was not significantly correlated with miR-339-3p and miR-361-5p expression (Additional file 6: Fig. S3B). Hence it was speculated that miR-1265 might be the upstream miRNA that regulate C2CD4A expression. Previous studies confirmed that miR-1265 was considered as a tumor suppressor in bladder cancer, glioma, and osteosarcoma [27, 28, 33]. Notably, the results of FISH assay using TMA showed that the expression of miR-1265 was higher in adjacent noncancer tissues than in paired CRC tissues (Fig. 5C). However, there was no obvious correlations between miR-1265 expression and different clinicopathological features (Additional file 7: Table S7), including the prognosis of CRC patients (data not shown). To explore whether miR-1265 inhibited the expression of C2CD4A, qRT-PCR and western blot analysis indicated that C2CD4A mRNA and protein expression was significantly downregulated in the miR-1265/mimics (Fig. 5D, E). However, miR-1265/inhibitor induced the opposite effects (Fig. 5D, E). Then, the 3’UTR of C2CD4A, which was predicted to interact with miR-1265, was cloned into a luciferase reporter vector (wild type, WT). Additionally, a reporter carrying two mutated miR-1265 binding sites was also created (Mutant) (Fig. 5F). Luciferase results indicated that inhibited luciferase activity in the cell lysates had transfected with miR-1265/mimics comparing to those transfected with the negative control mimics in the 293 T and RKO cells, and were of no effect on the RKO cells with Mutant vectors (Fig. 5G). However, the luciferase activity in the cell lysates was increased when transfected with miR-1265/inhibitor in the HCT8 cells, but did not strengthen luciferase activity in those with Mutant vectors (Fig. 5G). Then, ectopic expression of miR-1265 reduced the levels of p53 and Phosphorylation of p53 (Ser15) protein, and the opposite effects were observed when downregulation of miR-1265 (Additional file 9: Fig. S5E). The aforementioned consequences indicated that miR-1265 targeted directly to the 3’UTR of C2CD4A mRNA and suppressed C2CD4A mRNA translation in CRC.

### MiR-1265 inhibits cell growth and promotes apoptosis in *vitro*

To explore the possible underlying biological mechanism of miR-1265 function in CRC cells, the results from cell counting assays which included CCK-8 assays, clone formation assays and EdU assays demonstrated that miR-1265/mimics significantly inhibited the growth and proliferation of RKO cells (Additional file 8: Fig. S4A, B and C), while miR-1265/inhibitor promoted growth and proliferation of HCT8 cells (Additional file 8: Fig. S4A, B and C). Meanwhile, miR-1265/mimics increased the proportion of apoptosis cells in RKO (Additional file 8: Fig. S4D), while miR-1265/inhibitor decreased apoptosis cells in HCT8 (Additional file 8: Fig. S4D). In conclusion, miR-1265 had inhibited cell growth and promoted cell apoptosis in CRC.

### **Circ****SLC6A6**** is overexpressed in CRC**

**Fig. 6:**
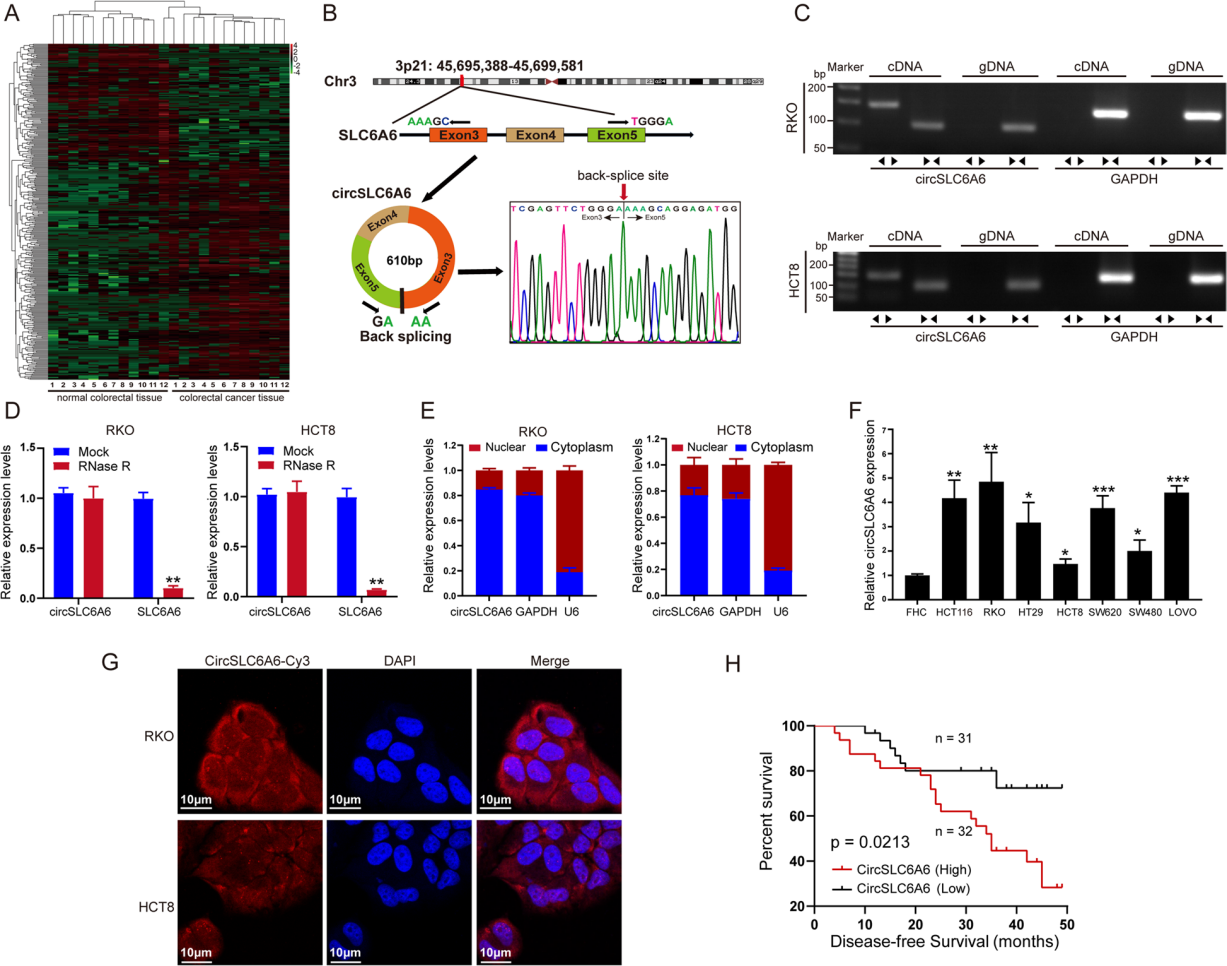
CircSLC6A6 is overexpressed in CRC. **a** The heatmap showed differentially expressed circRNAs form 12 paired fresh frozen CRC tissues in comparation with matched 12 adjacent normal mucosae tissues. **b** Schematic diagram showed the genomic loci of circSLC6A6. CircSLC6A6 was produced by 3-5 exons of SLC6A6; and the head-to-tail splicing junction of circSLC6A6 was confirmed by Sanger sequencing. **c** Divergent primer (circSLC6A6, ◀▶) and convergent primer (SLC6A6, ▶◀) were designed. The gel electrophoresis validated the existence of circSLC6A6. Divergent primers amplified circSLC6A6 in cDNA but not gDNA in RKO and HCT8 cells. GAPDH was used as a linear control. **d** The relative expression of circSLC6A6 and SLC6A6 mRNA in RKO and HCT8 cells were detected by qRT-PCR after the treatment of RNase R. **e** CircSLC6A6 was mainly located in the cytoplasm and determined by nuclear-cytoplasmic fractionation assay. **f** Relative expression of circSLC6A6 in CRC and FHC cell line were detected by qRT-PCR. **g** FISH assays helped to comfirmed that circSLC6A6 was mainly gathered in the cytoplasm of RKO and HCT8 cells. **h** Kaplan-Meier survival analysis (log-rank test) showed that CRC patients with high (32) circSLC6A6 expression were of lower DFS than that of low (31) expression of circSLC6A6. Using median circSLC6A6 expression as a cutoff value. All data are presented as the mean ± SEM (**P < 0.01, ***P < 0.001)

**Fig. 7:**
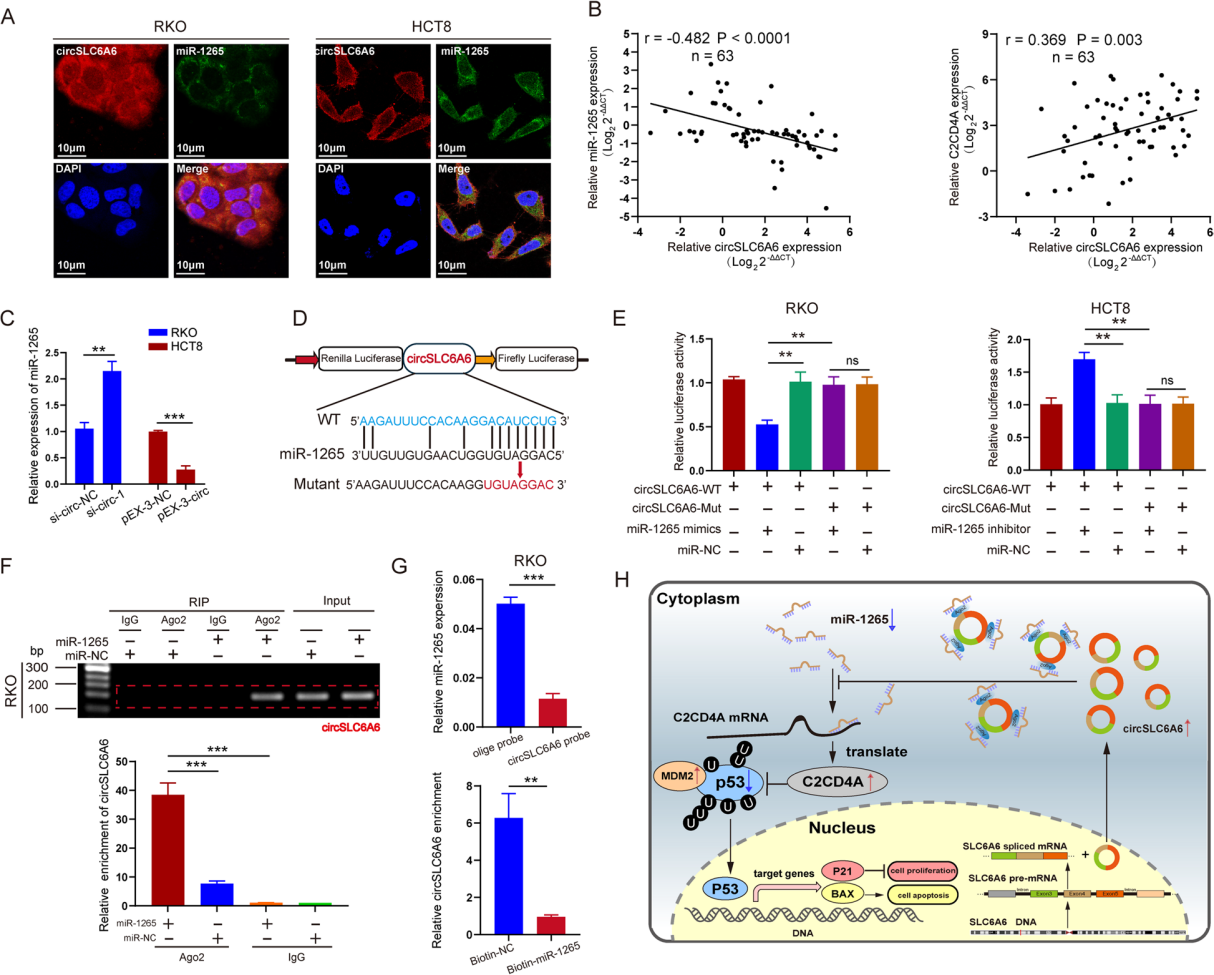
CircSLC6A6 functions as an efficient miR-1265 sponge, and alters the expression of C2CD4A through miR-1265 **a** Representative FISH images showed the co-location between circSLC6A6 and miR-1265 in RKO and HCT8 cells. **b** Correlations between circSLC6A6 with miR-1265 and C2CD4A mRNA expression were performed by Pearson’s correlation analysis in CRC tissue samples (n = 63). **c** qRT-PCR was applied to determine the effect of altered expression of circSLC6A6 on miR-1265 expression in RKO and HCT8 cells. **d** Putative binging sites between miR-1265 and circSLC6A6 were predicted by CircInteractome, and the schematic of circSLC6A6 wild-type (WT) and mutant (Mut) luciferase reporter vectors. **e** Luciferase activity of circSLC6A6-WT, circSLC6A6-Mut in RKO and HCT8 after co-transfection with miR-1265 cells mimics, inhibitor or miRNA control. **f** Anti-Ago2 RIP was performed in RKO cells transfected with miR-1265 mimics or miR-NC, then circSLC6A6 expression detected by agarose gel electrophoresis and qRT-PCR. **g** In RKO cells, endogenous miR-1265 was pulled down and enriched with circSLC6A6 probe, then the enrichment of miR-1265 was detected by qRT-PCR. Biotin-miR-1265 captured endogenous circSLC6A6 in the cell complex and was compared with biotin-NC then the enrichment of circSLC6A6 was detected by using qRT-PCR. Calculation of experiment results were followed by the ratio of pull-down to input. **h** Proposed model for circSLC6A6/miR-1265/C2CD4A/p53 axis regulatory network, circSLC6A6 as a ceRNA for miR-1265 to regulate C2CD4A and p53 expression in CRC. All data are presented as the mean ± SEM. (ns showed no significance. *P < 0.05, **P < 0.01, ***P < 0.001)

Accumulating studies confirmed that circRNAs was of great significance in tumor progression [34]. To identify the underlying dysregulated circRNAs in the CRC, RNA-seq analyses was performed to detect differentially expressed circRNAs between in 12 paired fresh frozen CRC tissues and corresponding adjacent normal colorectal mucosae tissues. We found that 373 differentially expressed circRNAs were identified with fold changes >2 or <0.5 (*P* < 0.05) (Fig. 6A). Differentially expressed circRNAs which included hsa_circ_0023984, hsa_circ_0008192, hsa_circ_0008694, hsa_circ_0004705 and hsa_circ_0001153 were highly expressed in CRC. Combined with CircInteractome database (https://circinteractome.nia.nih.gov/), bioinformatics analysis revealed that hsa_circ_0004705, termed as circSLC6A6, had possessed miR-1265 binding sites (Fig. 7D). Therefore, we focused on circSLC6A6, which was spliced from the SLC6A6 gene on chr3: 3p21: 45,695,388-45,699,581, with an ultimate length of 610 nucleotides. By comparing SLC6A6 mRNA sequences with the expected sequences of circSLC6A6 acquired from circBase (http://www.circbase.org/), schematic diagram illustrated that circSLC6A6 was originated from exons 3, 4 and 5 of its parental SLC6A6 pre-mRNA (Fig. 6B). It was further confirmed the splicing sites via Sanger sequencing (Fig. 6B). However, head-to-tail splicing could be the result of not only trans-splicing but also genomic rearrangements [35]. To mark off above two varieties, special divergent primers to amplify circSLC6A6 and convergent primers for circSLC6A6 mRNA were designed. cDNA and gDNA were extracted separately from RKO and HCT8 cells which were subjected to nucleic acid electrophoresis detection. It was indicated that hat circSLC6A6 could be detected only in cDNA (141-bp fragment), with no products were detected in the extracted gDNA (Fig. 6C). To verified the stability of circSLC6A6, total RNA treatment of RNase R. qRT-PCR results showed that RNase R failed to digest circSLC6A6, further confirming the circular structure of circSLC6A6 (Fig. 6D). Furthermore, the results of nuclear cytoplasmic fractionation illustrated that circSLC6A6 was mainly positioned in the cytoplasm (Fig. 6E). Next, circSLC6A6 was prominently expressed in CRC cell lines in comparation with FHC cell line via qRT-PCR. Among CRC cell lines, RKO showed the highest level of circSLC6A6, whereas HCT8 the lowest level (Fig. 6F). Additionally, the FISH results also showed that circSLC6A6 was located predominantly in the cytoplasm (Fig. 6G). All these results confirmed that circSLC6A6 was highly stable in cytoplasm of CRC cells. CircSLC6A6 was markedly upregulated in CRC tissues compared with adjacent non-tumor tissues (Additional file 9: Fig. S5A, B). More importantly, the patients with higher circSLC6A6 expression harbor a relatively worse DFS (Fig. 6I). All those findings suggested that circSLC6A6 was upregulated both in CRC cells and tissues and was a vital role in diagnosis and prognosis of CRC.

### **Circ****SLC6A6**** functions as an efficient miR-1265 sponge, and alters the expression of C2CD4A and p53 through miR-1265**

Numerous studies have reported that circRNAs might function as miRNA sponges to abrogate the functions of miRNAs [35, 36]. Hence, we explored whether circSLC6A6 promoted CRC growth and proliferation by sponging miRNAs. Firstly, by utilizing CircInteractome database, it was predicted that the potential target miRNAs of circSLC6A6 and the possible downstream miRNAs. It was indicted that miR-1265 shared binding site with circSLC6A6 attracted our attention. The FISH results revealed that co-location of circSLC6A6 and miR-1265 were mainly located in cytoplasm (Fig. 7A). In addition, the results from qRT-PCR indicated that circSLC6A6 expression was negatively correlated with miR-1265 expression, and positively associated with C2CD4A expression in 63 pairs CRC tissues (Fig. 7B), which indicated that circSLC6A6 might be involved in regulating miR-1265 and C2CD4A expression. All the results revealed that circSLC6A6 might interact with miR-1265 in CRC. To address the regulation of circSLC6A6 on miR-1265, three circSLC6A6-targeting siRNAs (si-circ-1, si-circ-2, si-circ-3) and a circSLC6A6 overexpression vector (pEX3-circ) were constructed to alter the expression of circSLC6A6. The result of qRT-PCR showed that the altered expression of circSLC6A6 had no significantly effect on the expression of SLC6A6 mRNA (Additional file 9: Fig. 54C, D). Then, we monitored the effect of altered circSLC6A6 expression on miR-1265 expression. Downregulation of circSLC6A6 could decrease miR-1265 expression and upregulation one could increase miR-1265 expression in RKO and HCT8 cells, respectively (Fig. 7C). Additionally, full length circSLC6A6 sequences (WT, wild type) and sequences with mutant binding site (Mutant) were constructed (Fig. 7D). Luciferase reporter assay results indicated that miR-1265/inhibitor relatively strengthened the luciferase activity of vector containing wild circSLC6A6 but did not alter the luciferase activity of the vector containing mutant circSLC6A6 (Fig. 7E). Conversely, miR-1265/mimics relatively subdued the luciferase activity of vector with wild circSLC6A6 but did not decrease the vector with mutant circSLC6A6 (Fig. 7E).

Next, RIP assays using anti-Ago2 or IgG were carried out in RKO cells with transiently transfected with miR-1265 mimics or controls, followed by agarose gel electrophoresis qRT-PCR and analysis for circSLC6A6 levels (Fig. 7F). The RIP results showed that circSLC6A6 pulled down with anti-Ago2 antibody was significantly enriched in RKO cells after being transfected with miR-1265 mimics compared to controls, suggesting that miR-1265 could directly target circSLC6A6 in AGO2 manner. Moerover, RNA pull-down assay indicated that endogenous miR-1265 or circSLC6A6 were significantly pulled down by biotinylated probes against miR-1265 or circSLC6A6, displaying the existence of circSLC6A6/miR-1265 complexes. The results indicated that specific overexpression miR-1265 was detected in the circSLC6A6 pull-down pellet. Moreover, more circSLC6A6 was captured in the biotin-miR-1265 groups than in the biotin-negative control (NC) groups (Fig. 7G), suggesting that circSLC6A6 served as an effient miR-1265 sponge. Moreover, downregulation of circSLC6A6 markedly attenuated the expression of C2CD4A and MDM2, and increased the expression of p53, p21, Bax, and MDM2 (Additional file 9: Fig. S5F). Conversely, upregulation of circSLC6A6 enhanced the expression of C2CD4A and MDM2, and decreased the expression of p53, p21, Bax, and MDM2, which indicated that circSLC6A6 positively regulated the expression of C2CD4A (Additional file 9: Fig. S5G) . However, miR-1265/inhibitor partly reversed the decreased expression C2CD4A induced by downregulated circSLC6A6 expression, and miR-1265/mimics reversed the overexpression of C2CD4A induced by upregulated circSLC6A6 expression, indicating that miR-1265 could reverse the regulation of circSLC6A6 on C2CD4A expression (Additional file 9: Fig. S5 F, G). Therefore, we demonstrated that circSLC6A6 acted as miR-1265 sponge and lessen the repressive effect of miR-1265 on C2CD4A and P53 expression, thereby upregulating C2CD4A and downregulating P53 expression. Collectively, these results unveiled a new mechanism that circSLC6A6 bind directly to miR-1265 and promote the proliferation of CRC through enhancing C2CD4A expression.

### **Circ ****SLC6A6****promotes cell growth and inhibits apoptosis in*vitro***

To investigate the biological functions of circSLC6A6 in CRC cells, results from cell proliferation assays, including CCK-8 assays, EdU assays and clone formation assays, demonstrated that downregulated circSLC6A6 significantly suppressed the growth and proliferation of RKO cells (Additional file 10: Fig. S6A, B and C), while the enforced expression of circSLC6A6 remarkably promoted the growth and proliferation of HCT8 cells (Additional file 10: Fig. S6A, B and C). Meanwhile, downregulated circSLC6A6 increased the rate of apoptosis cells in RKO (Additional file 10: Fig. S6D), while enforced expression of circSLC6A6 decreased the rate of HCT8 apoptosis cells (Additional file 10: Fig. S6D). Collectively, these findings suggested that circSLC6A6 promoted cell growth,and inhibited cell apoptosis in CRC.

## Discussion

In our present results, C2CD4A was significantly upregulated in CRC tissues and cells. To elucidate the mechanism of C2CD4A in resulting in CRC growth, we observed that C2CD4A integrated with p53 to facilitate ubiquitin degradation of p53 through increasing interaction of MDM2 with p53. Additionally, our results showed that circSLC6A6 regulated C2CD4A and p53 expression by sponging miR-1265 to promote growth of CRC cells. Our findings was the first to explore the function of circSLC6A6/miR-1265/C2CD4A/p53 axis in the growth of CRC.

C2CD4A, which usually conferring human diabetes susceptibility was firstly reported as a gene relevant to CRC in our study related to CRC. It was found that the knockdown or overexpression of C2CD4A was capable of inhibiting or facilitating the growth of CRC. To further reveal the roles of C2CD4A in CRC, our attention was focused on the downstream molecular mechanisms underlying C2CD4A. The expression of C2CD4A in RKO cells was knocked-down, and cDNA arrays were performed to screen for altered genes. According to the results of analysis of DEGs by KEGG analysis, we speculated that C2CD4A might promote the CRC growth and proliferation by repressing the p53 signaling pathway. IP/MS also confirmed that C2CD4A may interact with p53. It was being well acknowledged that ubiquitination-induced proteasomal degradation of p53 was essential to maintain p53 protein homeostasis, whose disruption was a molecular marker for cancer in recent 40 years [37, 38]. It was demonstrated by Wang et al. that TRIM67, a transcriptional target of p53, functioned as a tumor suppressor by directly binding with the C terminus of p53 and protecting it from MDM2-mediated ubiquitination in CRC [39]. Yet, the regulation aberrant expression of p53 protein had not been thoroughly studied [40]. In this study, we firstly described the aberrant upregulated C2CD4A in CRC tissue and its critical role in regulating p53 protein stability, C2CD4A regulated the expression of p53 target genes, including p21 and BAX. We also found that C2CD4A shortened half-life of p53, suggesting that C2CD4A might promote CRC oncogenesis through decreasing p53 function. Furthermore, p53 accumulation was enhanced in C2CD4A-silenced HCT116 p53^+/+^ cells. Next, we confirmed that C2CD4A, as a novel regulator of the p53 pathway, interacted with p53 and promoted p53 ubiquitination Thus, C2CD4A-mediated p53 degradation implied that C2CD4A might be critical for CRC growth and tumorigenesis. The E3 ubiquitin ligase MDM2 had acted as a vital negative regulator of p53 protein level and activity among plenty of proteins which involved in p53 regulation [24]. MDM2 was bound to p53 and ubiquitinates it proteasomal degradation. Intriguingly, the upregulated C2CD4A expression was able to increase the binding of MDM2 and p53 to facilitate p53 degradation. These results indicated that C2CD4A mediated p53 ubiquitin-degradation through increasing the interaction of MDM2 with p53. MDM2 may be a vital factor in C2CD4A mediated-p53 ubiquitination.

Numerous studies confirmed that aberrantly expressed miRNAs had regulated CRC development and progression [41, 42]. The biological function of miR-1265 widely considered as a tumor-suppressive role in several cancers and its molecular targets in the process of tumorigenesis had been illustrated in several studies [27, 28, 33]. In present study, we found that miR-1265 was significantly downregulated in CRC tissues. Subsequently, our study demonstrated that miR-1265 was directly bound to 3’UTR of C2CD4A to decrease C2CD4A expression and therefore inhibited the growth of CRC. Luciferase reporter assay also verified this direct interaction between miR-1265 and C2CD4A in CRC cells. Currently, circRNAs served as the most popular topic in the field of carcinogenesis and cancer progression [43]. Accumulating evidence disclosed that circRNAs were involved in the regulation of cancer cell proliferation, survival, migration and differentiation [13, 16, 44, 45]. Up till now, the miRNA sponge was still the most common mechanism for circRNA to exert its biological functions [13, 46]. Particularly, our previous study indicated that circMLLT10 served as a sponge of miR-509-3-5p, and promoted gastric cancer cell progression through increasing the expression of GINS4 and then activating Rac1 and CDC42 [44]. Moreover, our recently study demonstrated that circCCDC9 directly sponged miR-6792-3p to relieve the repressive effect of miR-6792-3p on its target CAV1, and then to suppress the progression of gastric cancer [17]. Circ-ITCH up-regulates p21 and PTEN through sponging miR-17 and miR-224, which suppressed the aggressive biological behaviors of bladder cancer [47]. In this study, it was identified that circSLC6A6 derived from SLC6A6 gene, which was highly expressed in CRC tissue and was capable of sponging miR-1265, and thus elevated C2CD4A expression and stimulated CRC growth and proliferation. There were several pieces of evidence implicating that circSLC6A6 functioned as a sponge of miR-1265 to regulate C2CD4A in CRC. First, circSLC6A6 expression negatively correlated with miR-1265, whereas positively correlated with C2CD4A expression in 63 fresh frozen CRC tissues. Second, downregulated circSLC6A6 would separately lead to a higher miR-1265 and lower C2CD4A expression and up-regulated circSLC6A6 resulted in opposite effects. Third, bioinformatics analyses and luciferase reporter assays verified this prediction. Fourth, miR-1265 inhibition reversed the effect of downregulated C2CD4A expression caused by knockdown of circSLC6A6, and overexpression of miR-1265 reversed the effect of upregulated C2CD4A expression caused by overexpression of circSLC6A6. Finally, circSLC6A6 could regulate C2CD4A expression, thus leading to the C2CD4A-reduced p53 degradation and inhibiting downstream pathway (Fig. 7H). In conclusion, circSLC6A6 was demonstrated to be an important candidate oncogene in CRC and was involved in the prognosis of CRC patients. CircSLC6A6 might act as a miR-1265 sponge to abolish the inhibitory effects on C2CD4A expression, which eventually promoted the growth of CRC cells. Our study revealed that the circSLC6A6/miR-1265/C2CD4A/p53 axis was involved in the pathogenesis and development of CRC, indicating that this axis might be a novel therapeutic target in patients with CRC.

## Conclusion

To conclude, for the first time, our results reveals that circSLC6A6/miR-1265/C2CD4A axis, which was involved in CRC growth via the p53 signaling pathway, may provide new ideas and targets for CRC treatment.

## Supplementary Information

**Additional file 1.**

**Additional file 2.**

**Additional file 3.**

**Additional file 4.**

**Additional file 5.**

**Additional file 6.**

**Additional file 7.**

**Additional file 8.**

**Additional file 9.**

**Additional file 10.**

## Data Availability

The data sets used in this study can be obtained from the corresponding author on reasonable request.

## Abbreviations

AJCC: American Joint Commission on Cancer
CHX: cycloheximide
CircRNA: Circular RNA
C2CD4A: C2 calcium dependent domain containing 4A
ceRNA: Competing endogenous RNA
CRC: Colorectal cancer
DEGs: differentially expressed genes
DFS: Disease-free survival
DOX: Doxorubicin
FISH: Fluorescence in situ hybridization
IHC: Immunohistochemistry
IP/MS: Immunoprecipitation and Mass Spectrometry
miRNA: MicroRNA
KEGG: Kyoto Encyclopedia of Genes and Genomes
qRT-PCR: Real-time quantitative polymerase chain reaction
OS: Overall survival
RIP: RNA immunoprecipitation
TMA: Tissue microarray
3’UTR: 3’-untranslated region
SIX1: Sine oculis homeobox homolog 1
SLC6A6: Solute carrier family 6 member 6
TRIM67: Tripartite motif containing 67
