# Supplementary material for: The novel circSLC6A6/miR-1265/C2CD4A axis promotes colorectal cancer growth by suppressing p53 signaling pathway
Source: J Exp Clin Cancer Res. 2021 Oct 16;40:324. doi: 10.1186/s13046-021-02126-y (PMC8520208; doi:10.1186/s13046-021-02126-y)
Supplement: Supplementary file 1 — Additional file 1. [file 13046_2021_2126_MOESM1_ESM.pdf]

Supplementary Materials and Methods

Cell culture and culture conditions

The human CRC (HCT116, RKO, HT29, HCT8, SW620, SW480, and LOVO), 293 T and normal colon epithelial (FHC) cell lines were purchased from the Chinese Academy of Sciences, Shanghai Institutes for Biological Sciences (Shanghai, China). HCT116 $p53^{+/+}$ and HCT116 $p53^{-/-}$ cells were obtained from American Type Culture Collection (ATCC, Maryland, USA). All these cell lines were maintained in DMEM medium supplemented with 10% FBS at standard culture conditions (5% CO2, 95% humidity and 37 °C).

RNA extraction, gDNA extraction, and quantitative real-time polymerase chain reaction (qRT-PCR)

Total RNA was extracted from human CRC cells lines and frozen tissues with RNAiso Plus reagent (Takara, Japan) in line with the manufacturer's protocol. Genomic DNA (gDNA) was extracted from tissues using Fast Pure Cell/Tissue DNA Isolation Mini Kit (Vazyme, DC102) in accordance with the manufacturer's protocol. Cytoplasmic and nuclear RNAs were separated using PARIS™ Kit (Invitrogen, USA) following the manufacturer's protocol. For circRNA and mRNA, reverse transcription was conducted using the PrimeScript RT Master Mix (Takara, Japan). For miRNA, reverse transcription was conducted using PrimeScript RT Reagent Kit (Takara, Japan) with corresponding stem-loop primers. qRT-PCR was conducted using SYBR Green Master Mix (Yeasen, China) in line with the manufacturer's protocol on Roche real-time PCR instrument (Roche Applied Science, USA). Human GAPDH served as internal control for circRNAs and mRNAs. U6 served as internal control for miRNAs. All primers were listed in Additional file 2: Table S1. The relative RNA expression levels were determined by the $2^{-\Delta\Delta CT}$ method. Each qRT-PCR experiment was tested in triplicate.

Nucleic acid electrophoresis and RNase R treatment

Nucleic acid electrophoresis and RNase R treatment were conducted as previously reported [1, 2] .

Transfection, oligonucleotides and plasmids

For lentivirus transfections, short hairpin RNA (shRNA) plasmid targeting human

C2CD4A and the control plasmid (sh-Ctrl) were inserted into a lentiviral vector (Genechem Shanghai, China). The sequences to knockdown human C2CD4A were showed in Additional file 2: Table S2. The human C2CD4A gene was cloned into pLVX plasmids (Genechem, Shanghai, China) to construct the C2CD4A overexpression vector. Then, the indicated cells with lentiviral transduction were selected with puromycin (Beyotime Biotechnology, China) for two weeks. The efficiency of C2CD4A knockdown and overexpression were verified by qRT-PCR and western blotting. Meanwhile, to regulate circSLC6A6, miR-1265, and MDM2 expression, oligonucleotides and plasmids were constructed. The siRNAs targeting circSLC6A6 and MDM2 were provided by GenePharma (Shanghai, China). The relevant oligonucleotides sequences were presented in Additional file 2: Table S2. Additionally, full length of circSLC6A6 was successfully cloned into the lentiviral plasmid pEX-3 (GenePharma, Shanghai, China) to establish the cell line with stable expression of circSLC6A6. The mimics, inhibitor and controls for miR-1265 were purchased from GenePharma (Shanghai, China). Cells transfection were performed using Lipofectamine 2000 (Invitrogen).

Immunohistochemistry (IHC)

The slides were incubated with primary antibodies including p53 (1:100, Abcam, USA), p21 (1:50, CST, USA), Cleaved-caspase3 (1:400; CST, USA), Bax (1:400, CST, USA), Ki-67 (1:500, Abcam, USA) at 4°C overnight, respectively, and then incubated with HRP labeled secondary antibody for 1 h at room temperature. The details about IHC staining score were shown in our previously published research [3].

Western blot analysis

All cells and tissues were lysed with RIPA lysis buffer (New Cell & Molecular Biotech Co, China). Total protein concentration was measured by BCA Protein Assay Kit (Beyotime Biotechnology, China). Protein lysates (50μg) were separated in different concentrations of sodium dodecyl sulfate-polyacrylamide gels electrophoresis (SDS-PAGE) and then transferred to PVDF membranes (Millipore, Billerica, USA). The membranes were subsequently blocked in 5% non-fat milk at room temperature for 2h and then incubated at 4 °C overnight with the primary antibodies: anti-C2CD4A (1:500; Sigma-Aldrich, USA. The applications not including IHC staining), anti-Flag (1:5000;

Sigma-Aldrich, USA), anti-His (1:1000; CST, USA), anti-HA (1:1000, CST, USA), anti-GFP (1:1000; Abcam, USA), anti-MDM2 (1:1000; CST, USA), anti-p53 (1:1000; Abcam, USA), anti-Phospho-p53 (Ser15) (1:1000; CST, USA), anti-p21 (1:1000; CST, USA), anti-Ki67 (1:5000; Abcam, USA), anti-Bax (1:1000; CST, USA), anti-Cleaved-caspase3 (1:1000; CST, USA), anti-GAPDH (1:1000; CST, USA). Then, the membranes were incubated with appropriate secondary antibodies (1:000; CST, USA) for 2h. After membranes being washed, the brands were detected using ECL chemiluminescent reagent (Millipore, MA, USA). GAPDH was used as a loading control.

For protein half-life detection, HCT116 $p53^{+/+}$ cells were respectively stably transfected with shC2CD4A-1, shC2CD4A-2 and pLVX-C2CD4A, and were treated with 10 mg/mL cycloheximide (CHX). Then, the treated cells were respectively harvested after CHX treatment for 0, 20, 40, 60, 90 and 120 min. Equal amount of protein from different samples was subjected to western blot analysis with anti-C2CD4A or anti-p53 antibody as indicated.

Cell proliferation assay

For the cell counting kit-8 (CCK-8) (Dojindo, Japan) assay, the transduced CRC cells were seeded in 96-well microplates with 1000 cells per well in 5% CO2 at 37°C. OD values at 450 nm were measured every 24 hours by Gen5 microplate reader (BioTek). For the 5-Ethynyl-2'-deoxyuridine (EdU) cell growth assay, the 2×10^4 CRC cells were seeded into 96-well microplates and cultured for 24h. Then, the cells were incubated with 50 μM EdU (RiboBio, Guangzhou, China) for 2h, then, cell nuclei were stained with Hoechst 33342 for 30 min. The percentage of EdU-positive cells was presented by: (EdU positive cells/Hoechst positive cells) ×100%. Colony formation assays were performed to evaluate the cloning capability of CRC cells, the transduced CRC cells were seeded into 6-well plates (2000 cells/well) and incubated at 37°C to facilitate colony formation. Two weeks later, the cells were fixed in methyl alcohol for 30min and then stained with 1% crystal violet for 20min. All experiments were performed in triplicate.

Apoptosis analysis

The cell apoptosis assay was conducted by using the AnnexinV-PE/7-AAD Apoptosis Detection Kit (MultiSciences Biotech Co, Ltd) in accordance with the manufacturer's protocol [3]. The apoptotic cells rate were detected and analyzed by a flow cytometrer (BD Biosciences, San Jose, CA, USA). All experiments were performed in triplicate.

The cancer genome atlas (TCGA) database, GEPIA dataset and Oncomine database

The CRC data was downloaded from TCGA database (https://cancergenome.nih.gov). There are 461 colon cancer samples with available data in TCGA database, including 458 RNA-seq samples, of which 41 pairs with paired sample data and pathological information. There are 171 rectal cancer samples with available data in TCGA database, of which RNA-seq samples have 166; there are 9 pairs with paired sample data and pathological information. Our expression profile analysis is based on these 50 paired samples with RNA-seq data, paired sample data and pathological information. In the above 50 pairs of CRC tissues and adjacent tissues, after filtering and screening, significantly differentially expressed genes were found. Gene Expression Profiling Interactive Analysis (GEPIA) dataset (http://gepia.cancer-pku.cn/detail.php) were used to determine the expression of C2CD4A in CRC tissues and normal tissues. Oncomine database (https://www.oncomine.org) was performed to detect the level of C2CD4A expression in CRC and normal adjacent tissues.

cDNA array analysis

cDNA array were performed as previously described to identify differentially expressed genes (DEGs) between three pairs RKO/sh-C2CD4A-1 and RKO/sh-Ctrl cells [4]. Then, Kyoto Encyclopedia of Genes and Genomes (KEGG) analysis were used to analyze the DEGs.

References

1. Luo Z, Rong Z, Zhang J, Zhu Z, Yu Z, Li T, et al. Circular RNA circCCDC9 acts as a miR-6792-3p sponge to suppress the progression of gastric cancer through regulating CAV1 expression. Mol Cancer. 2020;19(1):86.

2. Zhu Z, Rong Z, Luo Z, Yu Z, Zhang J, Qiu Z, et al. Circular RNA circNHSL1 promotes gastric cancer progression through the miR-1306-3p/SIX1/vimentin axis. Mol Cancer. 2019;18(1):126.
3. Rong Z, Luo Z, Zhang J, Li T, Zhu Z, Yu Z, et al. GINS complex subunit 4, a prognostic biomarker and reversely mediated by Kruppel-like factor 4, promotes the growth of colorectal cancer. Cancer Sci. 2020;111(4):1203-17.
4. Zhu Z, Yu Z, Rong Z, Luo Z, Zhang J, Qiu Z, et al. The novel GINS4 axis promotes gastric cancer growth and progression by activating Rac1 and CDC42. Theranostics. 2019;9(26):8294-311.
