# Supplementary material for: The novel circSLC6A6/miR-1265/C2CD4A axis promotes colorectal cancer growth by suppressing p53 signaling pathway
Source: J Exp Clin Cancer Res. 2021 Oct 16;40:324. doi: 10.1186/s13046-021-02126-y (PMC8520208; doi:10.1186/s13046-021-02126-y)
Supplement: Supplementary file 2 — Additional file 2. [file 13046_2021_2126_MOESM2_ESM.pdf]

Additional file 2

Table S1

The sequences of primers for qRT-PCR

	Sequence (5’-3’)
C2CD4A forward	TATTCCTCATCCTGCTTTTGG
C2CD4A reverse	CAGCTCATCTCAATGCCTG
p53 forward	CCTCAGCATCTTATCCGAGTGG
p53 reverse	TGGATGGTGGTACAGTCAGAGC
p21 forward	AGGTGGACCTGGAGACTCTCAG
p21 reverse	TCCTCTTGGAGAAGATCAGCCG
BAX forward	TCAGGATGCGTCCACCAAGAAG
BAX reverse	TGTGTCCACGGCGGCAATCATC
FAS forward	TGCCCAAGTGACTGACATCA
FAS reverse	CATCCCCATTGACTGTGCAG
TiGAR forward	TGCCACCTTTGGGATTC
TiGAR reverse	ATAGAGTGGTGAGGGTTGAG
SESN1 forward	TGCTTTGGGCCGTTTGGATAA
SESN1 reverse	TGTAGTGACGATAATGTAGGGGT
miR-1265 stem-loop	GTCGTATCCAGTGCAGGGTCCGAG GTATTCGCACTGGATACGACAACAAC
miR-1265 forward	GCGCAGGATGTGGTCAAGT
miR-1265 reverse	AGTGCAGGGTCCGAGGTATT
miR-339-3p stem-loop	GTCGTATCCAGTGCAGGGTCCGAGGTATT CGCACTGGATACGACCGGCTC
miR-339-3p forward	TGAGCGCCTCGACGACA
miR-339-3p reverse	AGTGCAGGGTCCGAGGTATT
miR-361-5p stem-loop	GTCGTATCCAGTGCAGGGTCCGAGGTATT CGCACTGGATACGACGTACCC
miR-361-5p forward	GCGCGTTATCAGAATCTCCAG
miR-361-5p reverse	AGTGCAGGGTCCGAGGTATT
circSLC6A6 forward	GTCATCGAGTTCTGGGAAAAGC
circSLC6A6 reverse	GGTTTTCCCTCAGCCTCGTC
SLC6A6 forward	GGTGTATGCCTTTTGTGGGTTG
SLC6A6 reverse	CATGTCCTCAATACCATCATAAAGG
U6 stem-loop	CTCAACTGGTGTCGTGGAGTCGGCAATT CAGTTGAGAAAAATAT
U6 forward	CAAGGATGACACGCAAA
U6 reverse	TCAACTGGTGTCGTGG
GAPDH forward	TGACTTCAACAGCGACACCCA
GAPDH reverse	CACCCTGTTGCTGTAGCCAAA

Table S2

	Sequence (5'-3')
The sequences to silence human C2CD4A	
sh-C2CD4A-1	GATGATTCTAGATTAACTATCT
sh-C2CD4A-2	GTCTAAAGGGCTCGCTATTAA
The sequences to silence human MDM2	
si-NC	GGCCAGGCTGCTAACGCACATGTCATA
si-MDM2	GTCGCGAGGGCTATGAACTAATGACCC
The sequences to silence circSLC6A6	
si-circ-1	UCGAGUUCUGGGAAAAGCATT
si-circ-2	GUUCUGGGAAAAGCAAGGATT
si-circ-3	GGGAAAAGCAAGGAGAUGGTT

Table S3

Probes used in this study.

FISH Probes	Sequence (5'-3')
Cy3-circSLC6A6	TTGCTTTTCCCAGAACTCGAT
FAM-miR-1265	AACAACACTTGACCACATCCTG
Cy3-miR-1265	AACAACACTTGACCACATCCTG

Table S4

Biotin-coupled probes	Sequence (5'-3')
Oligo probe	GTGTAACACGTCTATACGCCCA
Biotin-circSLC6A6	CTTGCTTTTCCCAGAACTCGA
Biotin-miR-1265	AACAACACTTGACCACATCCTG

Table S5

The relative mRNA expression changes of CDKN1A, BAX, Ki67, FAS, SESN1, TiGAR, and SERPINE2 from cDNA array data.

Gene Symbol	Regulation	Fold Change
CDKN1A(p21)	up	1.76845341
BAX	up	1.61633921
Ki-67	down	1.58901197
FAS	up	1.63055149
SESN1	up	1.74831684
TiGAR	down	1.61330961
SERPINE2	down	1.50571981
