# Supplementary material for: The novel circSLC6A6/miR-1265/C2CD4A axis promotes colorectal cancer growth by suppressing p53 signaling pathway
Source: J Exp Clin Cancer Res. 2021 Oct 16;40:324. doi: 10.1186/s13046-021-02126-y (PMC8520208; doi:10.1186/s13046-021-02126-y)
Supplement: Supplementary file 4 — Additional file 4. [file 13046_2021_2126_MOESM4_ESM.pdf]

Supplementary Figure. 1

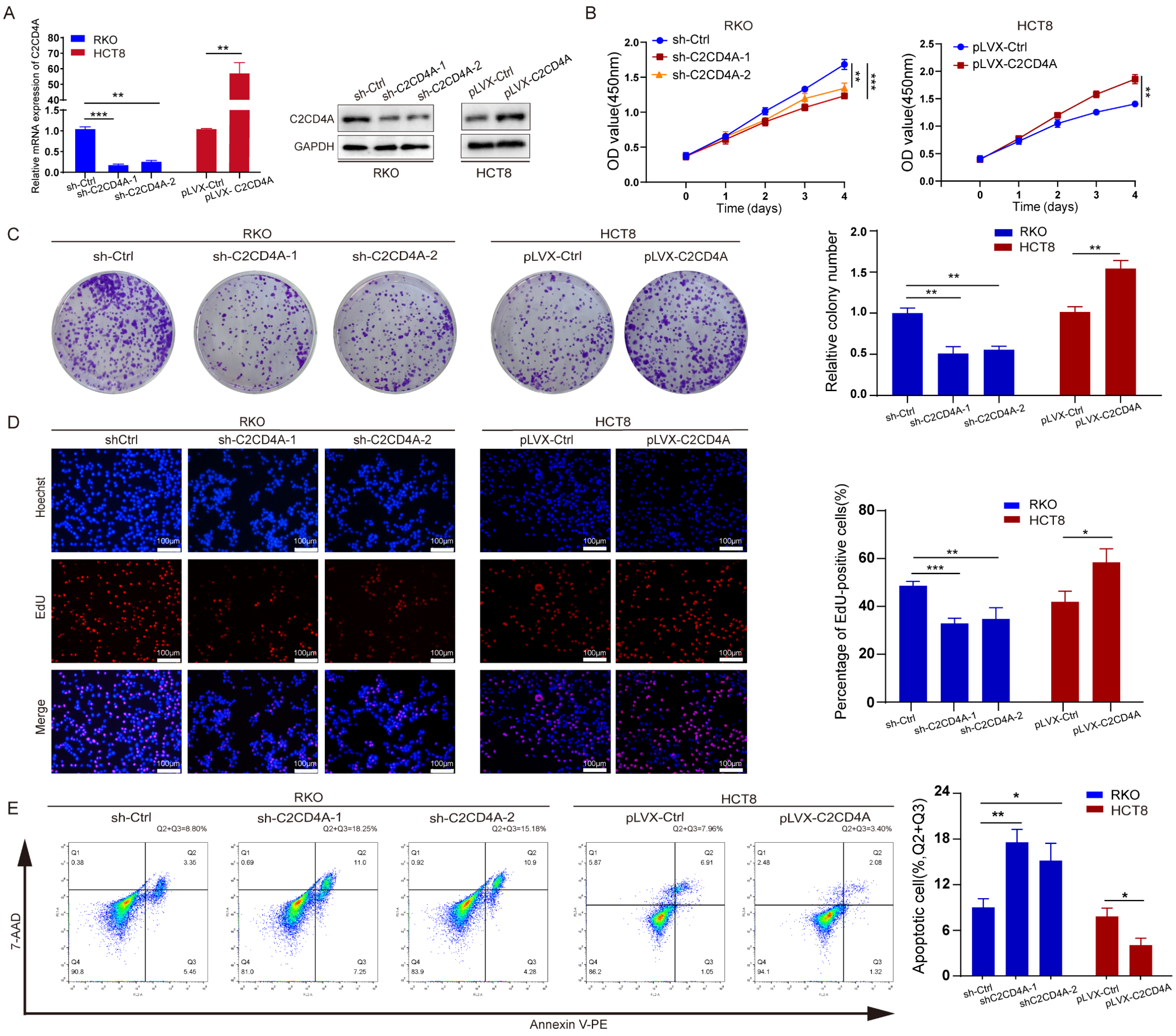

Figure. S1 C2CD4A promotes cell growth and inhibits apoptosis in *vitro*. a C2CD4A mRNA and protein expression in stable RKO and HCT8 cells were detected by qRT-PCR and western blot. **b** CCK-8 assay was applied to detect the effects of downregulation and upregulation of C2CD4A in CRC cells. **c** Colony formation assay helped to detect the effects of downregulation and upregulation of C2CD4A in CRC cells, the representative images and quantification results are shown. **d** Cell growth change caused by downregulation and upregulation of C2CD4A were detected by EdU assays. **e** Apoptosis analysis were used to detect the effects of downregulation and

upregulation of C2CD4A in CRC cells. Three independent experiments were performed for each group. (**$P < 0.01$, ***$P < 0.001$).
