# Supplementary material for: The novel circSLC6A6/miR-1265/C2CD4A axis promotes colorectal cancer growth by suppressing p53 signaling pathway
Source: J Exp Clin Cancer Res. 2021 Oct 16;40:324. doi: 10.1186/s13046-021-02126-y (PMC8520208; doi:10.1186/s13046-021-02126-y)
Supplement: Supplementary file 5 — Additional file 5. [file 13046_2021_2126_MOESM5_ESM.pdf]

Supplementary Figure. 2

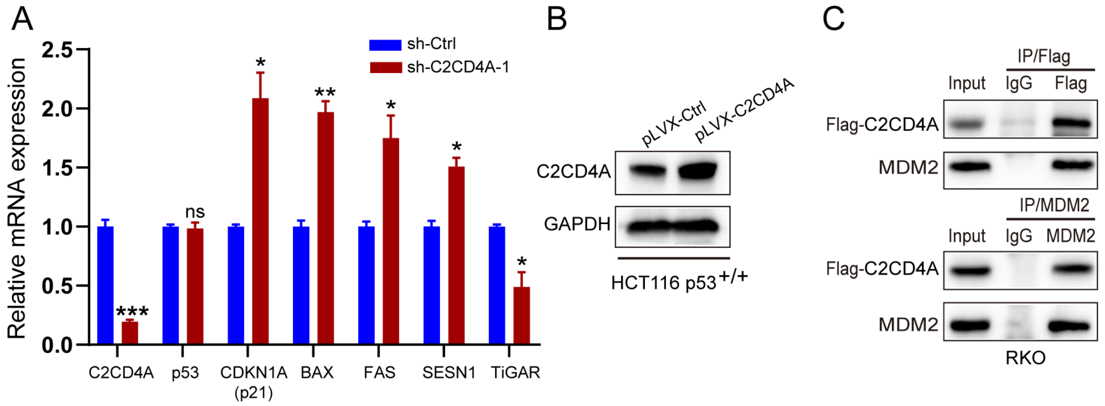

Figure. S2 a qRT-PCR was carried out to determine the relative mRNA expressions of C2CD4A, p53, CDKN1A(p21), BAX, FAS, and SESN1 in RKO cells. **b** Western blot was used to detect the expression of C2CD4A in HCT116 p53+/+ cells transfected with C2CD4A overexpression vector. **c** Co-IP assay analysis of the interaction between Flag-C2CD4A and MDM2 in RKO cells. (ns showed no significance. **P* < 0.05, ***P* < 0.01, ****P* < 0.001).
