# Supplementary material for: The novel circSLC6A6/miR-1265/C2CD4A axis promotes colorectal cancer growth by suppressing p53 signaling pathway
Source: J Exp Clin Cancer Res. 2021 Oct 16;40:324. doi: 10.1186/s13046-021-02126-y (PMC8520208; doi:10.1186/s13046-021-02126-y)
Supplement: Supplementary file 6 — Additional file 6. [file 13046_2021_2126_MOESM6_ESM.pdf]

Supplementary Figure. 3

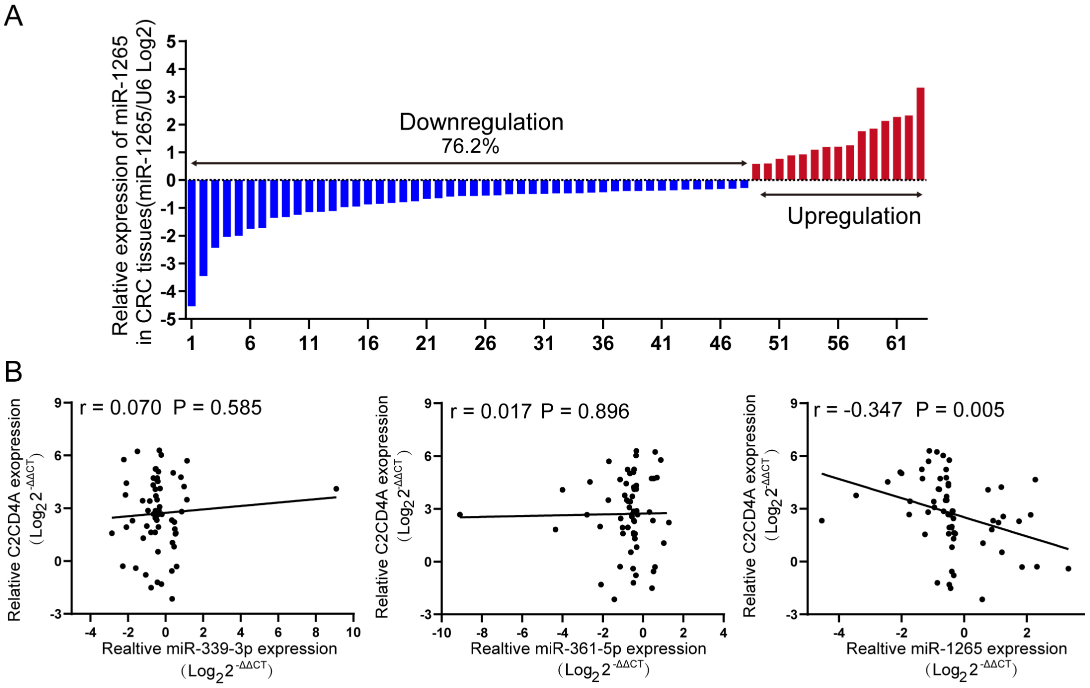

Figure. S3 a miR-1265 were observed to be upregulated in 48 (76.2%, 48/63) CRC tissues. **b** Correlations between miR-339-3p, miR-361-5p and miR-1265 with C2CD4A mRNA expression, were performed by Pearson's correlation analysis in CRC tissue samples (n=63), respectively.
