# Supplementary material for: The novel circSLC6A6/miR-1265/C2CD4A axis promotes colorectal cancer growth by suppressing p53 signaling pathway
Source: J Exp Clin Cancer Res. 2021 Oct 16;40:324. doi: 10.1186/s13046-021-02126-y (PMC8520208; doi:10.1186/s13046-021-02126-y)
Supplement: Supplementary file 7 — Additional file 7. [file 13046_2021_2126_MOESM7_ESM.pdf]

Table S7

Correlation between miR-1265 expression and clinicopathological parameters in CRC from TMAs

(n=106)

Characteristics		Case	miR-1265 expression			
			Low (73)	High (33)	χ^2	*P* value
Age						
	< 65	51	36	15	0.136	0.713
	≥ 65	55	37	18		
Gender						
	Male	55	42	13	2.996	0.083
	Female	51	31	20		
T stage						
	T1+T2	12	6	6	2.247	0.134
	T3+T4	94	67	27		
N stage						
	N0+N1	82	56	26	0.056	0.813
	N2+N3	24	17	7		
TNM stage						
	I＋II	51	35	16	0.003	0.959
	III＋IV	55	38	17		
Nerve invasion						
	Yes	27	17	10	0.054	0.816
	No	79	56	23		
Vessel invasion						
	Yes	40	32	8	3.713	0.054
	No	66	41	25		
Differentiation						
	Well	19	15	4	1.097	0.295
	Moderate+ Poor	87	58	29		
Tumor size						
	＜ 5cm	59	40	19	0.071	0.790
	≥ 5cm	47	33	14		
Tumor location						
	Right	41	31	10	1.417	0.234
	Left and Rectal	65	42	23		
