# Supplementary material for: The novel circSLC6A6/miR-1265/C2CD4A axis promotes colorectal cancer growth by suppressing p53 signaling pathway
Source: J Exp Clin Cancer Res. 2021 Oct 16;40:324. doi: 10.1186/s13046-021-02126-y (PMC8520208; doi:10.1186/s13046-021-02126-y)
Supplement: Supplementary file 8 — Additional file 8. [file 13046_2021_2126_MOESM8_ESM.pdf]

Supplementary Figure. 4 MiR-1265 inhibits cell growth and promotes apoptosis in *vitro*.

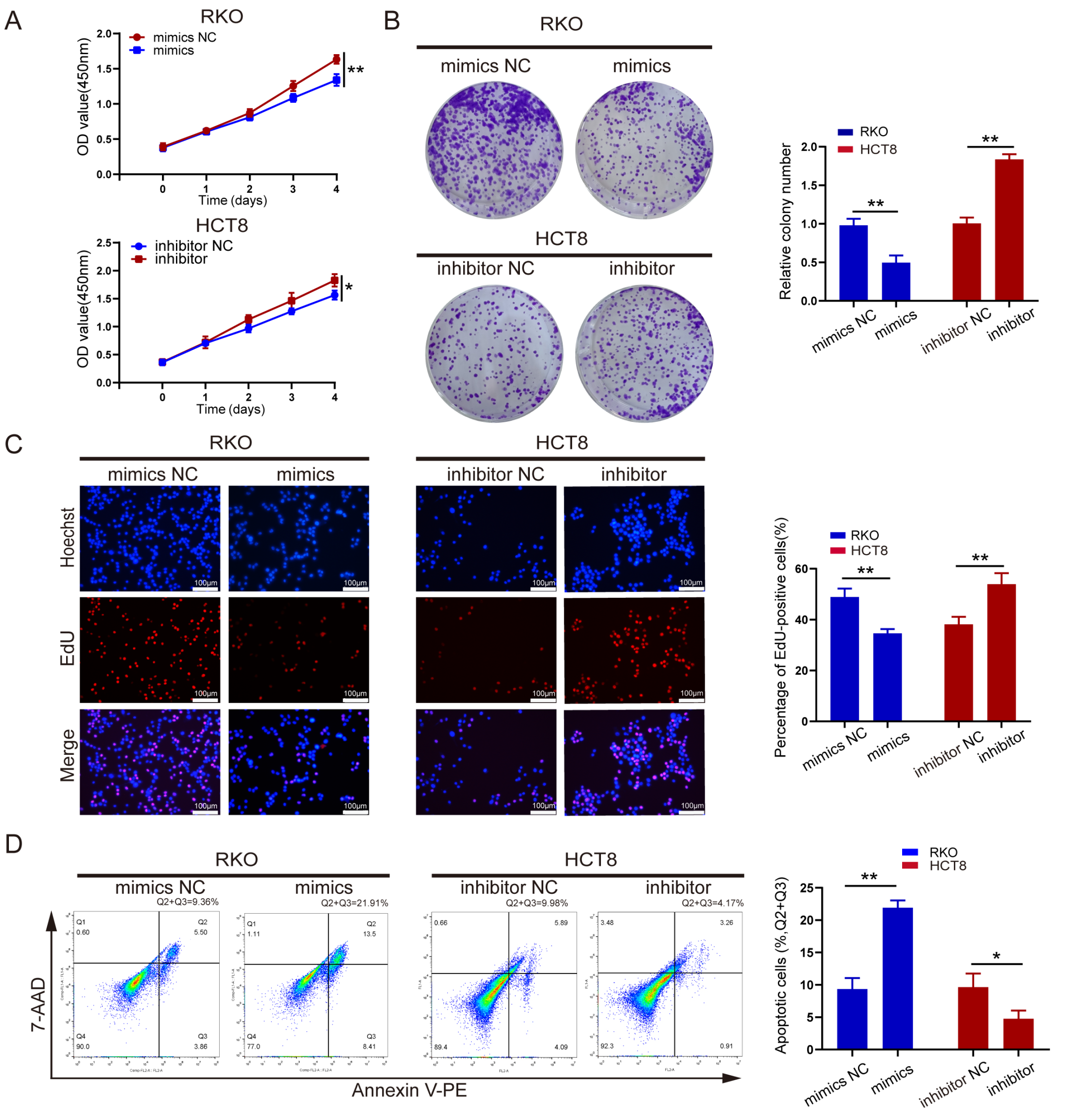

Figure. S4 MiR-1265 inhibits cell growth and promotes apoptosis in *vitro*. a CCK-8 assay was used to detect the effects of miR-1265/mimics and miR-1265/inhibitor in RKO and HCT8 cells. **b** Colony formation assay was used to detect the miR-1265/mimics and miR-1265/inhibitor in RKO and HCT8 cells. **c** Cell growth change caused by miR-1265/mimics and miR-1265/inhibitor were determined by EdU assays in RKO and HCT8 cells. **d** Apoptosis analysis were utilized to detect the effects of miR-1265/mimics and miR-1265/inhibitor in RKO and HCT8 cells. Three independent experiments were performed for each group (*$P < 0.05$, **$P < 0.01$).
