# Supplementary material for: The novel circSLC6A6/miR-1265/C2CD4A axis promotes colorectal cancer growth by suppressing p53 signaling pathway
Source: J Exp Clin Cancer Res. 2021 Oct 16;40:324. doi: 10.1186/s13046-021-02126-y (PMC8520208; doi:10.1186/s13046-021-02126-y)
Supplement: Supplementary file 9 — Additional file 9. [file 13046_2021_2126_MOESM9_ESM.pdf]

Supplementary Figure. 5

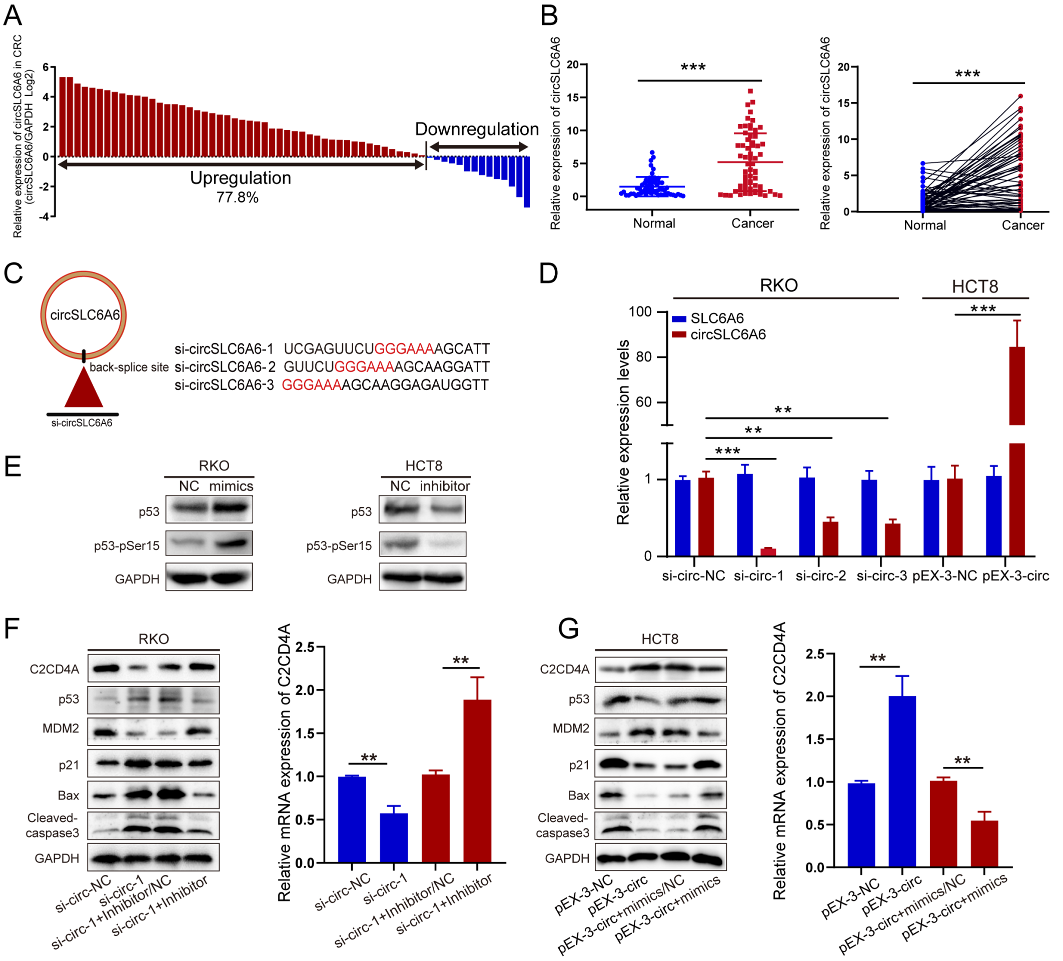

Figure. S5 a, **b** CircSLC6A6 expression was higher in CRC tissues than in adjacent normal tissues. **c** Schematic representation of three si-RNA targeting different sites of circSLC6A6. **d** The effects of circSLC6A6 knockdown and overexpression were determined by qRT-PCR. **e** Western blot analysis of p53 and p53-pSer15 in RKO and HCT8 cells with knockdown or overexpression of miR-1265. **f, g** qRT-PCR and western blot were used to measure the C2CD4A, p53, p21, Bax, Cleaved-caspase3 in RKO and HCT8 cells. All data were presented as the mean ± SEM (***P* < 0.01, ****P* < 0.001).
