# Supplementary material for: The novel circSLC6A6/miR-1265/C2CD4A axis promotes colorectal cancer growth by suppressing p53 signaling pathway
Source: J Exp Clin Cancer Res. 2021 Oct 16;40:324. doi: 10.1186/s13046-021-02126-y (PMC8520208; doi:10.1186/s13046-021-02126-y)
Supplement: Supplementary file 10 — Additional file 10. [file 13046_2021_2126_MOESM10_ESM.pdf]

Supplementary Figure. 6 CircSLC6A6 promotes cell growth and inhibits apoptosis in *vitro.*

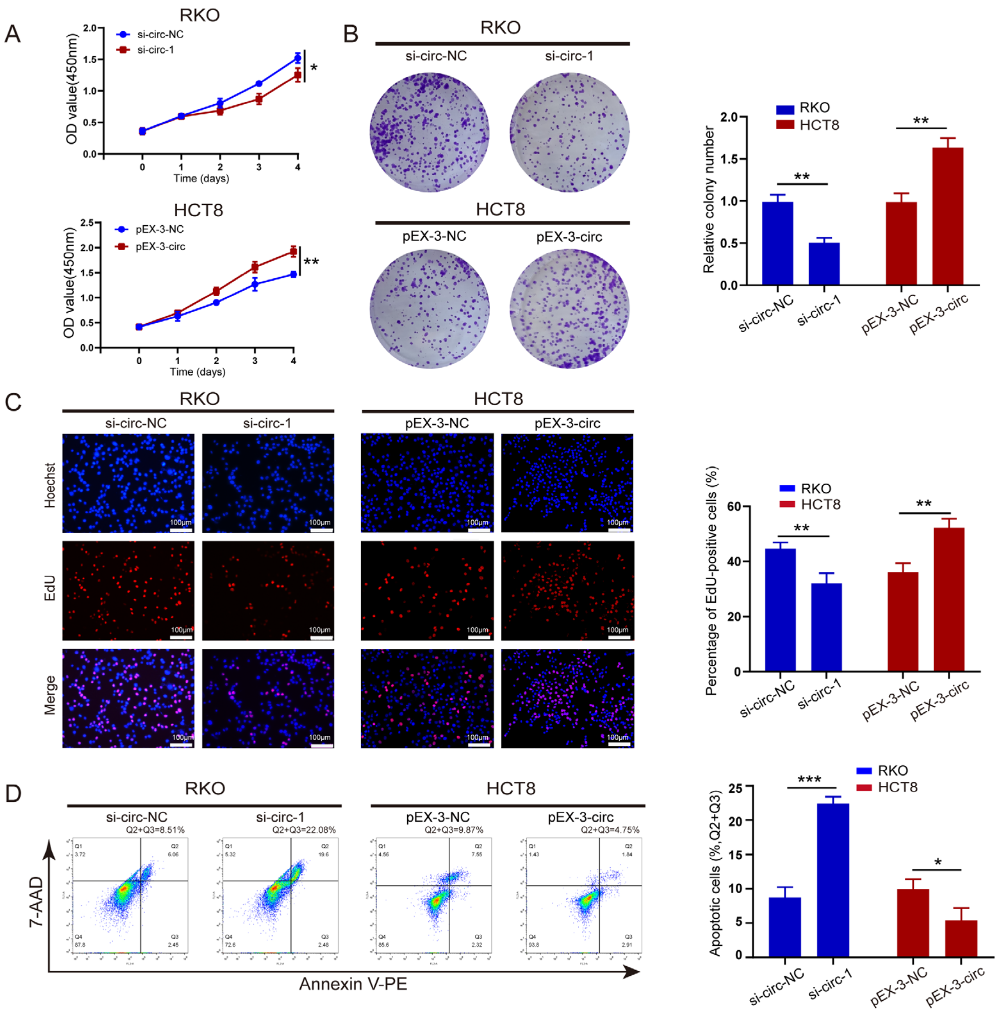

Figure. S6 CircSLC6A6 promotes cell growth and inhibits apoptosis in *vitro.* a CCK-8 assay was used to detect the effects of downregulation and upregulation of circSLC6A6 in RKO and HCT8 cells. **b** Colony formation assay was used to detect the effects of downregulation and upregulation of circSLC6A6 in RKO and HCT8 cells. **c** The changes in cell growth caused by downregulation and upregulation of circSLC6A6 were detected by EdU assays in RKO and HCT8 cells. **d** Apoptosis analysis were used to detect the effects of downregulation and upregulation of circSLC6A6 in RKO and HCT8 cells. Three independent experiments were performed for each group (*$P < 0.05$, **$P < 0.01$).
